# Context-Dependent IL-1 mRNA-Destabilization by TTP Prevents Dysregulation of Immune Homeostasis Under Steady State Conditions

**DOI:** 10.3389/fimmu.2020.01398

**Published:** 2020-07-07

**Authors:** Lucy Sneezum, Kevin Eislmayr, Helene Dworak, Vitaly Sedlyarov, Anita Le Heron, Florian Ebner, Irmgard Fischer, Yoichiro Iwakura, Pavel Kovarik

**Affiliations:** ^1^Max Perutz Labs, Vienna Biocenter, University of Vienna, Vienna, Austria; ^2^Research Institute for Biomedical Sciences, Tokyo University of Science, Tokyo, Japan

**Keywords:** mRNA stability, tristetraprolin, TTP, Zfp36, Interleukin 1beta, Interleukin 1alpha, inflammation, immune homeostasis

## Abstract

The bioavailability of the major pro-inflammatory cytokines IL-1α and IL-1β is tightly controlled by transcription and post-translational processing to prevent hyperinflammation. The role of mRNA decay in maintenance of physiological IL-1 amounts remained unknown. Here we show that the down-regulation of *Il1a* and *Il1b* mRNA by the mRNA-destabilizing protein TTP (gene *Zfp36*) is required for immune homeostasis. The TTP deficiency syndrome, a multi organ inflammation in *TTP*^−/−^ mice, was significantly ameliorated upon deletion of the IL-1 receptor. *Il1a* and *Il1b* played non-redundant roles in triggering the pathological IL-1 signaling in *TTP*^−/−^ mice. Accordingly, tissues from *TTP*^−/−^ animals contained increased amounts of *Il1b* mRNA. Unexpectedly, TTP destabilized *Il1b* mRNA in cell type-specific ways as evident from RNA-Seq and mRNA stability assays. These results demonstrate that TTP-driven mRNA destabilization depends on the cellular context. Moreover, such context-defined mRNA decay is essential for keeping steady state IL-1 levels in the physiological range.

## Introduction

IL-1α and IL-1β are potent pro-inflammatory cytokines and thus subject to tight multilayered control (1, 2). They are encoded by the *Il1a* and *Il1b* genes, respectively, located on chromosome 2 in both humans and mice (1). Binding of IL-1α or IL-1β to the IL-1 receptor, composed of IL-1R1 (gene name *Il1r1*) and IL-1R3 (*Il1rap*), triggers a MyD88-dependent kinase cascade that culminates in activation of several transcription factors, such as NF-κB and AP1, and subsequent gene expression changes (1, 3). The response to IL-1 signaling comprises the induction of multiple cytokines, chemokines and prostaglandins which in turn regulate both innate and adaptive immunity. IL-1β is the most studied member largely because its uncontrolled production is associated with autoimmune diseases, non-resolving inflammation and cancer (4, 5). IL-1β promotes host defense against infection (6). IL-1α, the less understood IL-1 family member, is generally regarded as an important driver of sterile inflammation following tissue injury and cell damage (7). Thus, it can act as a danger signal, i.e., a DAMP (danger-associated molecular pattern). IL-1 signaling is regulated at the organismal level by IL-1α and IL-1β bioavailability, a process shown to involve transcription, post-translational cleavage, and liberation from the cell as revealed by a number of recent studies (reviewed in (7–10)). In contrast, studies addressing regulation of IL-1 cytokines at the level of their mRNA stability are scarce (11, 12), and the relevance of such regulation for IL-1 bioavailability and function has so far not been addressed.

mRNA decay plays a fundamental role in the control of inflammatory responses and maintenance of immune homeostasis (13, 14). The inflammatory response is causally associated with stabilization of intrinsically unstable, inflammation-induced mRNAs, in particular those of cytokines and chemokines, allowing rapid and efficient production of inflammatory mediators. The resolution phase of inflammation is characterized by a timely destabilization and degradation of these inflammation-induced mRNAs; this results in a swift shut down of cytokine production and re-establishment of homeostasis (14–16). mRNA stability is controlled by *cis*-acting regulatory elements, mostly located in the 3' untranslated regions (UTRs), as well as by RNA-binding proteins (RBPs) and miRNAs (14, 16).

Tristetraprolin (TTP), encoded by the gene *Zfp36*, is an mRNA-destabilizing protein that preferentially binds to AU-rich elements (AREs) in the 3′ UTRs (17). TTP restricts expression of the target mRNAs by enhancing their decay and, by poorly understood ways, inhibiting their translation (17–19). mRNA destabilization by TTP includes the recruitment of the CCR4-NOT deadenylase and the decapping complex, while inhibition of translation is less well-understood (17).

We previously reported a PAR-iCLIP and systems biology approach to map and functionally annotate TTP binding sites in the transcriptome of primary macrophages (i.e., cells relevant for TTP function)–the TTP Atlas (20). This study demonstrated that TTP binding to the 3' UTR of target mRNAs is essential but not sufficient for transcript destabilization since many mRNAs bound by TTP were stable. Thus, the study revealed the fundamental question about the identity of additional factors determining mRNA destabilization by TTP (20). Similar conclusions were also drawn by studies employing other cell systems (18, 21, 22).

In agreement with the role of TTP in destabilization of inflammatory mRNAs, *TTP*^−/−^ mice are born healthy but develop progressive and eventually lethal, pleiotropic inflammation: the so called “TTP deficiency syndrome” (23). The phenotype is likely caused by elevated levels of certain TTP target mRNAs; however the mechanistic understanding of the TTP deficiency phenotype remains a challenging area of research. The deletion of the TTP targets *Tnf* or *Il23* in *TTP*^−/−^ mice abolishes most of the TTP deficiency syndrome (23, 24). Nevertheless, the requirement for these cytokines does not exclude a causative role of other TTP targets which might act upstream or downstream of *Tnf* or *Il23*, or independently of them. The incompletely resolved mechanism causing the TTP deficiency syndrome is underlined by a study wherein the ARE, i.e., the TTP binding site, is removed from the *Tnf* locus: animals with this lesion spontaneously develop colitis which is a pathological condition not detected in *TTP*^−/−^ mice (25). Moreover, the cell type(s) conferring the TTP phenotype are not well-characterized. Bone marrow transplantation analyses suggested that *TTP*^−/−^ myeloid cells caused the TTP deficiency syndrome (26). However, follow-up studies by us and the Blackshear laboratory demonstrated that mice bearing myeloid cell-specific *TTP* deletion (LysMcre-*TTP*^fl/fl^) remained healthy (27, 28). A recent report showing that keratinocyte-specific *TTP*^−/−^ deletion recapitulates many, although not all, TTP deficiency symptoms (29) suggests that the phenotype of *TTP*^−/−^ mice is mechanistically more complex than originally anticipated. *TTP*^−/−^ mice are not fertile reflecting their poor health conditions (23).

Here, we employed *TTP*^−/−^ mice which were lacking also the IL-1 receptor (*TTP*^−/−^ *Il1r1*^−/−^), IL-1α (*TTP*^−/−^ *Il1a*^−/−^), or IL-1β (*TTP*^−/−^ *Il1b*^−/−^) to show that IL-1 cytokines significantly contribute to development of the TTP deficiency syndrome: *TTP*^−/−^ *Il1r1*^−/−^ mice were fertile, displayed normal weight gain and reduced inflammation of joints and dermis. In agreement with the hyperactivation of IL-1 receptor signaling in *TTP*^−/−^ mice, *Il1b* expression was upregulated in tissues of *TTP*^−/−^ mice. Detailed studies including RNA-Seq and mRNA decay assays using primary cells showed that *Il1b* mRNA is destabilized by TTP. Unexpectedly, *Il1b* mRNA was destabilized in a context-dependent way: *Il1b* was destabilized by TTP in dendritic cells but not macrophages. The TTP deficiency syndrome was marginally improved in *TTP*^−/−^ *Il1a*^−/−^ and *TTP*^−/−^ *Il1b*^−/−^ mice indicating that both IL-1α and IL-1β contribute to the phenotype of *TTP*^−/−^ mice. Together, this report defines IL-1 as an important driver of the TTP deficiency syndrome and identifies context-dependent mRNA destabilization as a process required for the maintenance of immune homeostasis.

## Materials and Methods

### Mice

WT, *TTP*^−/−^, *Il1r1*^−/−^ *TTP*^−/−^, *Il1a*^−/−^ *TTP*^−/−^, and *Il1b*^−/−^ *TTP*^−/−^ mice were bred and kept in isolated, ventilated cages under specific pathogen-free conditions. *Il1r1*^−/−^ *TTP*^−/−^ mice were obtained by crossing *TTP*^−/−^ mice (23) with *Il1r1*^−/−^ mice (30). *Il1a*^−/−^ *TTP*^−/−^ mice were obtained by crossing *TTP*^−/−^ mice with *Il1a*^−/−^ mice (31); *Il1b*^−/−^ *TTP*^−/−^ mice were obtained by crossing *TTP*^−/−^ mice with *Il1b*^−/−^ mice (31). All mice used were on a C57BL/6 genetic background. Animal experiments were performed in accordance with the Austrian law for animal experiments and approved by the Austrian Ministry of Science under the license number BMWFW-66.006/0019-WF/V/3b/2016.

### Cell Culture

Bone marrow-derived dendritic cells (BMDCs) and macrophages (BMDMs) were produced from bone marrow isolated from the femurs and tibias of 9–12 weeks old WT, *TTP*^−/−^ or *Il1r1*^−/−^ *TTP*^−/−^ mice. BMDCs were cultivated for 11 days in RPMI (Sigma) supplemented with 10% FCS (Sigma), 100 U/ml penicillin (Sigma), 100 μg/ml streptomycin (Sigma) and 200 U/ml GM-CSF (Peprotech). BMDMs were cultivated for 8 days in DMEM (Sigma) supplemented with 10% FCS (Sigma), 100 U/ml penicillin (Sigma), 100 μg/ml streptomycin (Sigma) and CSF-1 derived from L929-cells.

### RNA-Seq

BMDCs (12.5 × 10^6^ cells per 15 cm dish per sample) were left untreated or stimulated with 10 ng/ml LPS (Sigma) for 3, 6, or 9 h in biological triplicates. For total RNA extraction, 1 ml QIAzol Lysis Reagent (QIAGEN) was added. RNA was precipitated using isopropanol and GlycoBlue (Thermo Scientific). DNA digestion was performed using DNase (Roche). For library preparation, SENSE mRNA-Seq Library Prep Kit V2 (Lexogen) was used according to the manufacturer's protocol. The RNA quality was assessed using Agilent RNA 6000 Pico Assays that were analyzed on Agilent 2100 Bioanalyser. The sequencing was performed at the VBCF NGS Unit (https://www.viennabiocenter.org/facilities). Single-end fragment libraries (100 bp) were sequenced on the Illumina HiSeq 2500 platform. Raw sequencing reads were demultiplexed, and cutadapt (https://doi.org/10.14806/ej.17.1.200) was used for barcode, adapter and quality trimming. Quality control was performed using FastQC (http://www.bioinformatics.babraham.ac.uk/projects/fastqc/). STAR version 2.5 (32) was used to map all remaining reads to the GRCmm38/mm10 mouse genome assembly. We obtained more than 85% uniquely mapped reads in each sample. Data analysis and visualizations were performed in R-project version 3.4.0 with Rstudio IDE version 1.0.143. Differential expression analysis was performed based on read counts using DESeq2 (33). Gene set enrichment analysis (GSEA) was performed using the GSEA preranked module on the GenePattern server (34).

### Characterization of Primary Cells *in vitro*: Quantitative Reverse Transcription-PCR (qRT-PCR), Western Blotting and ELISA

BMDCs or BMDMs were treated with 10 ng/ml LPS (Sigma) for 3, 6, or 9 h. For induction of IL-1β release, 10 mM nigericin (Sigma) was added to cells for 2 h. IL-1α and IL-1β were added to cells at a concentration of 10 ng/ml for 3, 6, or 9 h. RNA isolation was performed as described for RNA-Seq, and cDNA was synthesized using Reverse Transcriptase (Thermo Scientific) with oligo (dT)_18_ and random hexamer primers. qRT-PCRs were run on a Realplex Mastercycler (Eppendorf) and monitored by the SYBR Green method using Hot FIREPol EvaGreen qPCR Supermix (Medibena). mRNA levels were calculated by the relative quantification method using a 2-fold standard dilution series derived from the samples. *Hprt* levels were used for normalization. For the purpose of analyzing mRNA decay rates, 5 μg/ml actinomycin D (Sigma) was added to cells for 45 or 90 min. Remaining mRNA was quantified using qRT-PCR, and the half-lives of transcripts then calculated using a fitted exponential curve. The 95% confidence intervals were calculated in R-project version 3.4.0.

For the purpose of Western blotting, whole cell extracts were prepared by lysing cells for 5 min in Frackleton buffer [10 mM Tris-HCl, 30 mM Na_4_P_2_O_7_, 50 mM NaCl, 50 mM NaF, 1% Triton X-100, 1 mM DTT, 1 mM vanadate, and 1x protease inhibitor (Roche)]. After centrifugation at 10,000 rpm at 4°C for 10 min, supernatants were added to SDS loading buffer at a 2:1 (lysate:loading buffer) ratio and boiled at 95°C for 5 min. For detection of TTP, MK2 and p-MK2, proteins were separated by SDS-PAGE on a 10% separation gel, and transferred to a nitrocellulose membrane by semi-dry transfer (Biorad Transblot® Turbo). For detection of IL-1β, proteins were separated by SDS-PAGE on a 12.5% separation gel, and transferred to a PVDF membrane by wet transfer. Anti-TTP (28), IL-1β (R&D Systems, #AF-401-NA), MK2 (Cell Signaling Technologies, #3042), p-MK2 (Cell Signaling Technologies, #3007) and tubulin (Cell Signaling Technologies, #2144S and Sigma, #T9026) antibodies were used for western blotting. Western blot quantification was performed on BioRad Image Lab 5.2.1.

Cytokine concentrations of IL-1β were measured in supernatants using a DuoSet ELISA kit (R&D Systems). For measurement of caspase-1 activity levels, the Caspase-Glo 1 Inflammasome Assay (Promega) was performed. Both were used according to the manufacturer's protocol.

### Phenotypic Characterization of Mice

WT, *TTP*^−/−^, *Il1r1*^−/−^ *TTP*^−/−^, *Il1a*^−/−^ *TTP*^−/−^, and *Il1b*^−/−^ *TTP*^−/−^ mice were phenotypically characterized for symptoms related to the TTP deficiency syndrome. Mice were weighed weekly up until 30 weeks of age and in parallel monitored for clinical signs of arthritis, dermatitis and conjunctivitis. Incidence of these inflammatory symptoms was defined as the first time any visual evidence of them was observed: arthritis by swelling of the paws and digits; dermatitis by drying and hardening of the paws or ears; and conjunctivitis by swelling of the conjunctiva.

For histological analysis of paw inflammation, front paws of mice were fixed in 4% paraformaldehyde for 16 h and decalcified for 10 days in Osteosoft (Merck Millipore). The tissue was then dehydrated and embedded in paraffin, and 4 μm sections prepared using a microtome. H&E staining was carried out following standard protocols. Sections were blind scored for the thickness of the epidermis and immune cell infiltrate in the dermis on a scale from 1 to 4, and on the degree of myeloid hyperplasia on a scale of 1–3, where 1 represents a WT phenotype and 3 or 4 a severe TTP deficiency phenotype.

Computer tomography (CT) imaging and measurements of bone volume and surface area/bone volume were performed at the VetCore Facility for Research (https://www.vetmeduni.ac.at/de_de/vetomics/). For measurement of degree of splenomegaly, 10-week-old mice were weighed and subsequently sacrificed, spleens isolated and weighed, and spleen weight normalized to total body weight.

### Cytokine Expression *in vivo*

Mesenteric lymph nodes, a 1 cm^2^ section of dorsal skin, the whole spleen, the liver left lobe and the right lungs were isolated from mice. For analysis of tissue mRNA expression levels, tissues were homogenized on a benchtop homogeniser (Polytron PT 2100) in QIAzol Lysis Reagent (QIAGEN) and RNA isolation, reverse transcription and qRT-PCR were performed as described for *in vitro* analyses. For analysis of tissue protein expression levels, tissues were homogenized in PBS supplemented with 1x protease inhibitor (Roche) and cytokine concentrations of IL-1α, IL-1β, and TNF-α were measured using DuoSet ELISA kits (R&D) according to the manufacturer's protocol. The determined concentrations were normalized to total protein concentration of the tissues as determined by Pierce BCA Protein Assay (Thermo Scientific) which was performed according to the manufacturer's protocol.

### Fluorescence Activated Cell Sorting (FACS) of Spleen Dendritic Cells/Monocytes

Spleens were isolated from 6 to 12 weeks old WT or TTP^−/−^ mice and single-cell suspensions prepared by plunging the cut spleens through 70 μm cell strainers. The cell suspensions were subsequently centrifuged at 500 × g for 5 min followed by lysis of red blood cells for 5 min in ACK buffer (Ammonium-Chloride-Potassium buffer: 150 mM NH_4_Cl, 10 mM KHCO_3_, 0.1 mM Na_2_EDTA, pH 7.3). After two washes in PBS, the isolated splenic cells were counted. From each spleen, 50 × 10^6^ cells were stained with Fixable Viability Dye eFlour 780 (eBioscience, #65-0865) prior to FcR blocking with anti-CD16/32 antibody (Biolegend, #1013) and stained in FACS buffer (PBS supplemented with 5% BSA and 5 mM EDTA) with the following monoclonal antibodies against mouse cell surface epitopes: anti-CD11c BV421 (Biolegend, #1173), anti-CD11b-BV605 (BD Biosciences, #563015), anti-Ly6G FITC (BD Biosciences, #551460), anti-Ly6C PerCP-Cyanine5.5 (eBioscience, #45-5932), anti-CD45R (B220) PE-Cyanine7 (BD Biosciences, #552772), anti-I-A/I-E (MHCII) PE (BD Biosciences, #557000), anti-CD3e PE-Cyanine7 (eBioscience, #25-0031). After staining, cells were washed twice with FACS buffer, resuspended in PBS and sorted using FACS Aria III flow cytometer operated with FACSDiva (BD Biosciences) as described in Supplemental Figure 6. DCs (cDCs and pDCs) were defined as CD11b^+^ CD11c^+^, monocytes as CD11b^+^ MHCII^−^ Ly6C^high^ and monocyte-derived DCs as CD11b^+^ MHCII^+^ Ly6C^−^ cells. Following sorting, RNA isolation was performed as described for RNA-Seq and reverse transcription performed using SuperScript IV Reverse Transcriptase (Thermo Fischer) following the manufacturer's protocol.

### qRT-PCR Primers

Il1b fw AGATGAAGGGCTGCTTCCAAA
Il1b rv AATGGGAACGTCACACACCA
Il1a fw CCA TCC AAC CCA GAT CAG CA
Il1a rv GTT TCT GGC AAC TCC TTC AGC
Tnf fw GATCGGTCCCCAAAGGGATG
Tnf rv CACTGGTGGTTTGCTACGAC
Hprt fw GCAGTCCCAGCGTCGTGA
Hprt rv CAGGCAAGTCTTTCAGTCCTGTC.

### Statistical Analysis

All statistical analysis was performed using GraphPad Prism 8.3.1.

## Results

### Regulation of the Transcriptome of Bone Marrow-Derived Dendritic Cells (BMDCs) by TTP

TTP regulates mRNA stability hence expression levels of predominately inflammation-associated genes such as cytokines and chemokines (20, 27, 28, 35, 36). Apart from this common signature TTP appears to regulate various mRNAs in cell type-specific ways as revealed by our recent analysis of distinct effects of TTP on the transcriptomes of two myeloid cell types, i.e., neutrophils and bone marrow-derived macrophages (BMDMs) (37). However, more detailed analysis of the cell type-specific impact of TTP was missing; in particular it was unclear whether the distinct effects of TTP on gene expression in different cells was caused indeed by differences in TTP-driven mRNA destabilization. Moreover, TTP displayed the highest impact on the neutrophil transcriptome prior to immunostimulation with lipopolysaccharide (LPS), which is in contrast to macrophages in which the effects of TTP were strongly increased upon LPS stimulation (20, 27, 28, 37).

**Figure 1:**
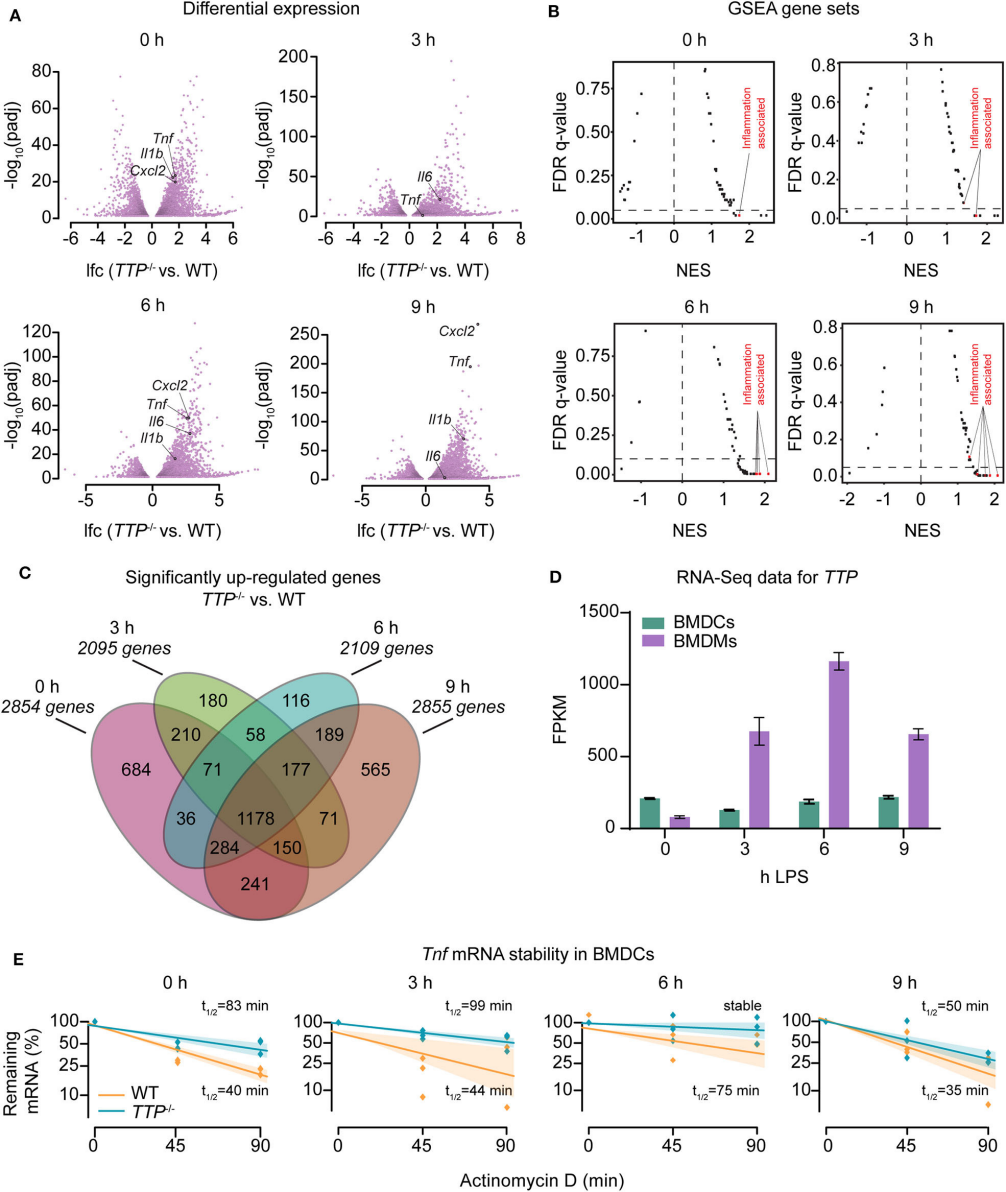
Regulation of the transcriptome of bone marrow-derived dendritic cells (BMDCs) by TTP. Bone marrow-derived dendritic cells (BMDCs) from WT and *TTP*^−/−^ mice were left untreated (0 h) or stimulated with 10 ng/ml LPS for 3, 6, and 9 h followed by RNA-Seq analysis. **(A)** Differential expression analysis between *TTP*^−/−^ vs. WT BMDCs. Only genes exhibiting significantly different expression (padj ≤ 0.05) are shown. Examples of cytokine and chemokine genes (*Tnf* , *Cxcl2, Il6*, and *Il1b*) are highlighted. **(B)** Mustache plot of false discovery rate (FDR) vs. normalized enrichment score (NES) based upon GSEA of RNA-Seq data. Dashed line: 0.05 FDR cutoff. **(C)** Overlap of genes significantly up-regulated (padj ≤ 0.05, LFC > 0) in *TTP*^−/−^ BMDCs vs. WT BMDCs at 0, 3, 6, and 9 h of LPS stimulation. **(D)** RNA-Seq data (FPKM, fragments per kilobase of exon model per million reads mapped) for *TTP* mRNA expression during LPS stimulation of WT BMDCs and, for comparison, WT BMDMs. RNA-Seq data for BMDMs are from our previous report (20). **(E)** *Tnf* mRNA stability assay performed by treating BMDCs with actinomycin D to block transcription and measuring remaining mRNA levels at 0, 45, and 90 mins. Shaded bars represent 95% confidence intervals. Half-lives (t_1/2_) are indicated.

To investigate the cell type- and immunostimulation-dependent impact of TTP further, we completed RNA-Seq experiments using dendritic cells as representative of another major myeloid cell type. Bone marrow-derived dendritic cells (BMDCs) from WT or *TTP*^−/−^ mice were treated with LPS (3, 6, or 9 h) or left untreated followed by RNA-Seq. Western blot analysis confirmed that BMDCs derived from *TTP*^−/−^ mice did not express TTP protein (Supplemental Figure 1A). Principal component analysis (PCA) of biological RNA-Seq triplicates confirmed that WT and *TTP*^−/−^ samples formed well-separated clusters (Supplemental Figure 1B). Differential expression analysis showed that TTP deficiency resulted in significantly increased (padj ≤ 0.05) expression of many genes under all conditions, as expected (Figure 1A; Supplemental Table 1). Gene set enrichment analysis (GSEA) revealed that the most highly upregulated genes were inflammation-associated genes (e.g. *Tnf* , *Il6*, and *Cxcl2*) supporting the well-characterized model of TTP-regulated gene expression (Figures 1A,B; Supplemental Table 1). However, the impact of TTP deficiency in BMDCs did not increase upon LPS stimulation, in contrast to BMDMs reported by us previously (20). The highest numbers of upregulated genes (padj ≤ 0.05, log_2_-fold-change [lfc] >0) in BMDCs were identified prior to stimulation and at 9 h of stimulation while the numbers of upregulated genes at 3 and 6 h of stimulation were 25% lower (Figure 1C). Thus, the contribution of LPS to TTP effects in BMDCs resembled the TTP impact in neutrophils but not BMDMs (20, 37). The analysis of TTP target genes revealed a major overlap under all conditions: 1178 genes upregulated in TTP-deficient BMDCs were shared across all time points of LPS treatments (i.e., 40–60% of genes upregulated in any condition) indicating that the TTP target spectrum was similar regardless of whether and how long the cells were treated with LPS (Figure 1C).

TTP mRNA expression in BMDCs remained largely unchanged upon LPS treatment contrasting a strong induction by LPS in BMDMs (Figure 1D). Consistently, TTP protein was expressed prior to LPS stimulation and the levels did not considerably change throughout the LPS time course in BMDCs while it was strongly induced by LPS in BMDMs (Supplemental Figure 1C). These data demonstrate different regulation of TTP by LPS in BMDCs vs. BMDMs and suggest that expression of TTP in BMDCs is regulated largely by intrinsic rather than extrinsic cues like LPS. We then asked whether the differences in TTP expression are accompanied by changes in phosphorylated (i.e., activated) MAPKAPK2 (MK2), a kinase known as important regulator of TTP expression. MK2 phosphorylates TTP at least on two residues (S52 and T178 in the mouse TTP) causing its stabilization and thereby increased expression (38, 39). In agreement, MK2 was phosphorylated in BMDCs but not in BMDMs prior to LPS treatment (Supplemental Figure 1D).

*Tnf* mRNA, the most extensively studied TTP target, has been shown to be destabilized by TTP in various cells (17). mRNA stability assays performed in BMDCs demonstrated that the decay rate of *Tnf* mRNA is reduced in the absence of TTP in both steady state (i.e., unstimulated) and inflammatory conditions (Figure 1E). Thus, TTP regulates the stability of *Tnf* mRNA in BMDCs as it does in many other cell types.

Collectively, the transcriptome-wide differential expression analysis revealed a significant impact of TTP on the transcriptome of dendritic cells. Unexpectedly, LPS did not result in a significant increase of TTP expression and TTP-dependent differential gene expression changes thereby contrasting the responses in macrophages. These data indicate that both TTP expression and TTP activity are regulated in cell type-specific ways.

### TTP Has Distinct Effects in BMDCs and BMDMs

**Figure 2:**
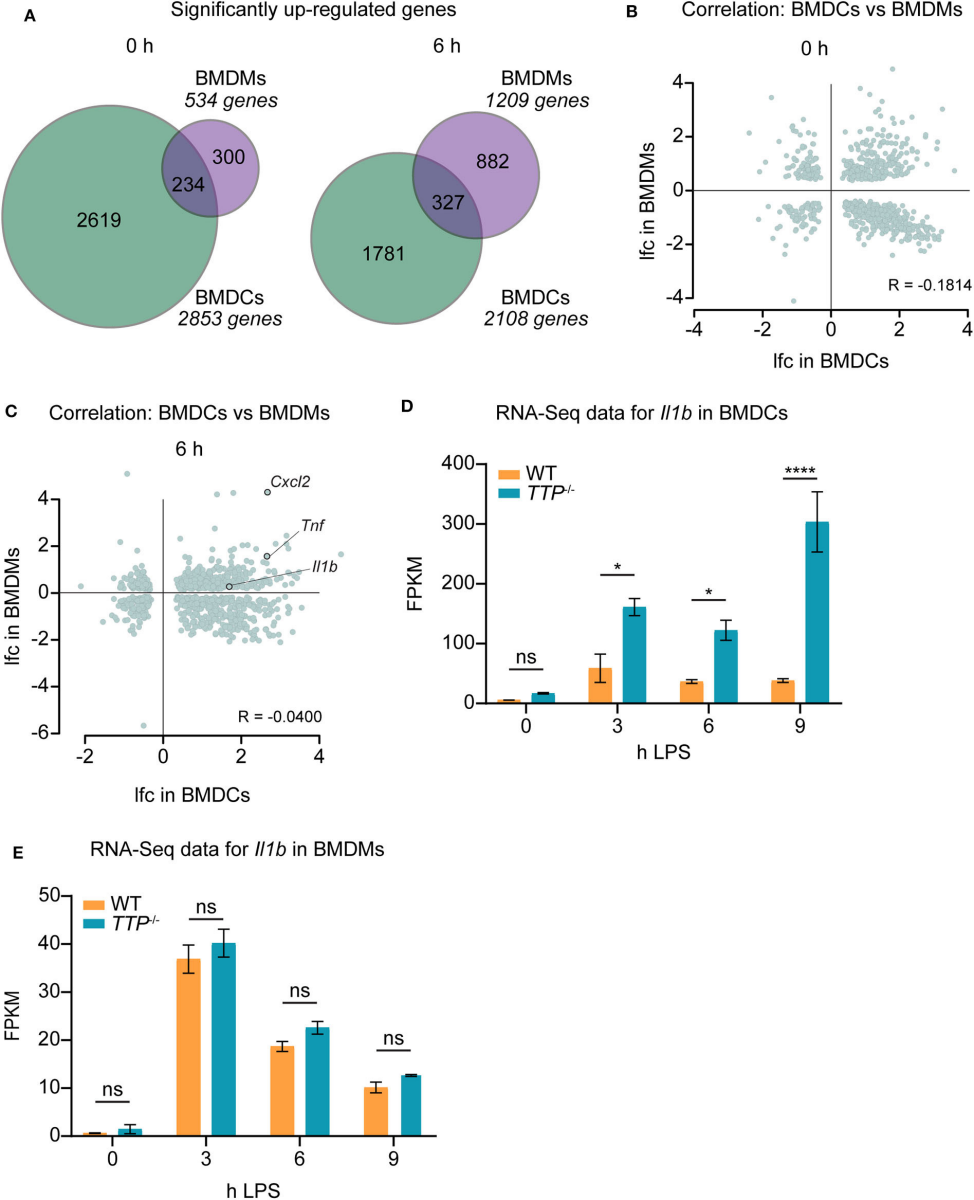
TTP has distinct effects in BMDCs and BMDMs. Differential expression data of RNA-Seq for *TTP*^−/−^ and WT BMDCs were compared with similar data previously obtained for BMDMs (20). **(A)** Overlap of significantly up-regulated (*TTP*^−/−^ vs. WT; padj ≤ 0.05, lfc > 0) genes in BMDMs and BMDCs at 0 and 6 h of LPS stimulation. **(B,C)** Correlation analysis for genes showing significantly different expression (padj ≤ 0.05) in BMDMs vs. BMDCs prior to LPS stimulation **(B)** and at 6 h of LPS stimulation **(C)**. lfc; log_2−_fold-change. *R*; Pearson's *R* correlation coefficient. The correlation coefficients *R* = −0.1814 and *R* = −0.0400 indicate that differential expression data for BMDMs do not correlate with those for BMDCs under any of the conditions. **(D)** RNA-Seq data (FPKM) for *Il1b* mRNA in *TTP*^−/−^ and WT BMDCs. **(E)** RNA-Seq data (FPKM) for *Il1b* mRNA in *TTP*^−/−^ and WT BMDMs as obtained in our previous report (20). Statistical evaluation in **(D,E)**: two-way ANOVA with Sidak's multiple comparisons test; error bars indicate mean ± SEM; *P < 0.05; ****P < 0.0001; ns, not significant.

The different regulation of TTP expression by LPS in BMDCs and BMDMs (Figure 1D) prompted us to compare the effects of TTP on the transcriptomes of these two myeloid cell types. To this end, we generated overlaps of genes upregulated (padj ≤ 0.05, lfc > 0) in the absence of TTP in BMDCs vs. BMDMs (Figure 2A). More genes were differentially regulated in BMDCs than in BMDMs under unstimulated as well as LPS-stimulated conditions. The difference was particularly pronounced prior to LPS stimulation with 5-fold more genes upregulated in BMDCs. Relatively few upregulated genes were shared between BMDCs and BMDMs (Figure 2A). These differences in TTP effects were confirmed by the absence of correlation between upregulated genes in BMDCs vs. BMDMs: *R* (Pearson's correlation coefficient) in unstimulated cells = −0.1814, *R* in stimulated cells = −0.0400 (Figures 2B,C).

The mRNAs of the pro-inflammatory genes *Tnf* and *Cxcl2* were highly upregulated in both cell types under LPS-stimulated conditions, i.e., under conditions of high expression of pro-inflammatory genes. Both *Tnf* and *Cxcl2* mRNAs are known to be bound and destabilized by TTP (20, 23, 28). Our data show that these two mRNAs are regulated similarly by TTP in BMDCs and BMDMs (Figure 2C, Supplemental Table 1). In contrast, the mRNA of the important pro-inflammatory cytokine *Il1b* was strongly upregulated in BMDCs but not BMDMs indicating different regulation of this gene in these two myeloid cell types (Figure 2C; Supplemental Table 1). Comparison of RNA-Seq data at all time points of LPS stimulation [BMDCs: Supplemental Table 1, BMDMs: (20)] confirmed that *Il1b* mRNA is regulated by TTP throughout the inflammatory response in BMDCs but not BMDMs (Figures 2D,E). We note that *Il1b* mRNA was bound by TTP in BMDMs as revealed by the analysis of the reported PAR-CLIP dataset (20) (Supplemental Figure 2). Thus, binding of TTP to *Il1b* mRNA does not regulate this target in BMDMs.

These data identify a distinct impact of TTP on gene expression in macrophages vs. dendritic cells. Together with the reported transcriptome-wide analysis of TTP impact in neutrophils (20), these results corroborate the cell type-specific effects of TTP within the myeloid lineage.

### TTP Controls *Il1b* mRNA Stability and IL-1β Protein Levels in BMDCs

**Figure 3:**
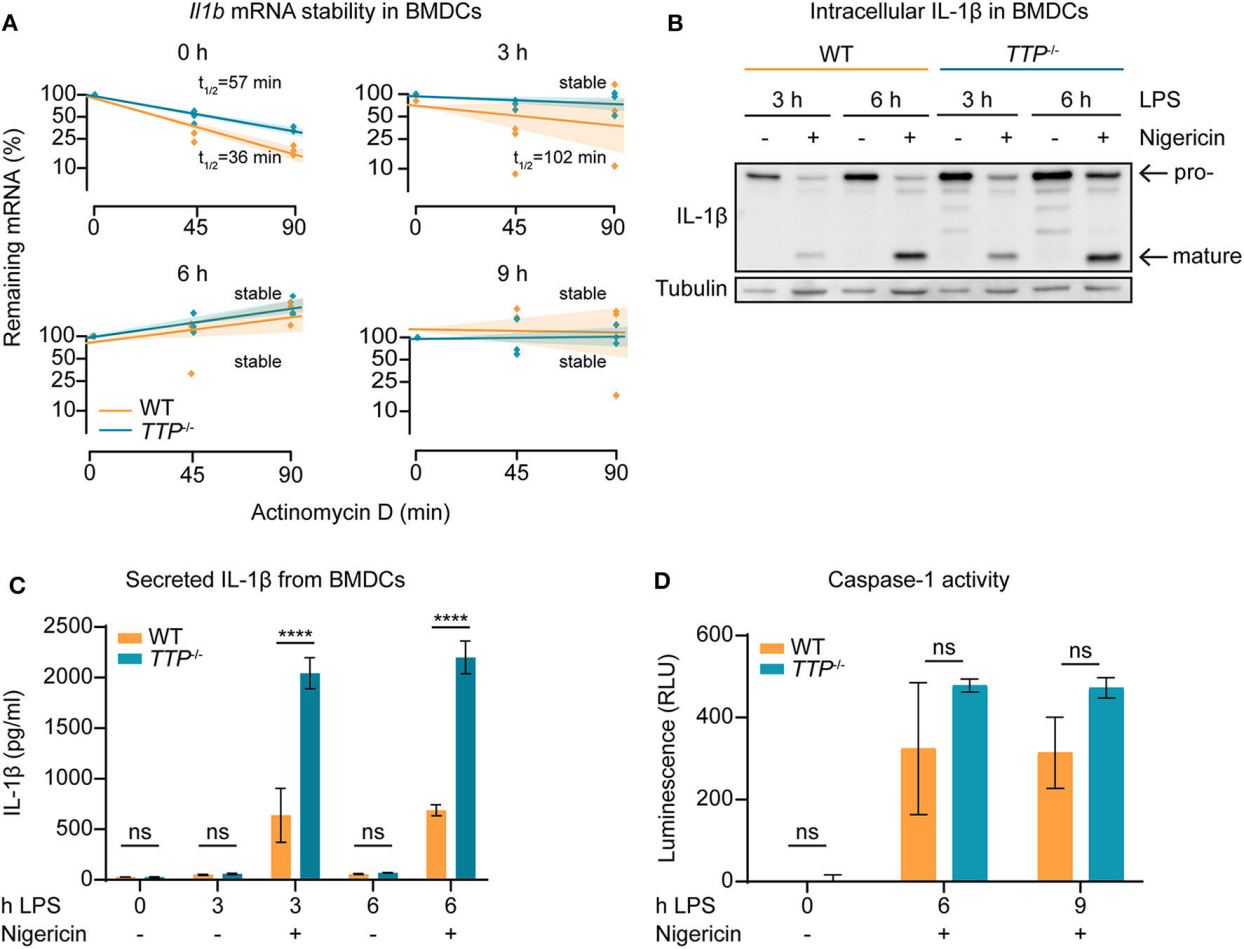
TTP controls *Il1b* mRNA stability and IL-1β bioavailability in BMDCs. **(A)** *Il1b* mRNA stability assay performed by treating cells with actinomycin D to block transcription and measuring remaining mRNA levels at 0, 45, and 90 mins using *TTP*^−/−^ and WT BMDCs stimulated for 0, 3, 6, and 9 h with LPS. Shaded bars represent 95% confidence intervals. Half-lives (t_1/2_) are indicated. **(B)** Western blot analysis for IL-1β using whole cell extracts from *TTP*^−/−^ and WT BMDCs stimulated for 3 and 6 h with LPS in the presence or absence of nigericin, as indicated. Pro-IL-1β and mature IL-1β forms are indicated. Tubulin served as loading control. **(C)** Secreted levels of IL-1β measured by ELISA using supernatants from *TTP*^−/−^ and WT BMDCs stimulated for 0, 3, and 6 h with LPS in the presence or absence of nigericin, as indicated. **(D)** Caspase-1 activity assessed by Caspase-1 Glo assay in *TTP*^−/−^ and WT BMDCs stimulated for 0, 6, and 9 h with LPS in the presence nigericin; RLU, relative luminescence units. Statistical evaluation in **(C,D)**: two-way ANOVA with Sidak's multiple comparisons test (*n* = 3); error bars, mean ± SEM; ****P < 0.0001; ns, not significant.

The increased *Il1b* mRNA levels in *TTP*^−/−^ BMDCs as compared to WT cells (Figure 2D; Supplemental Table 1) suggested that TTP promotes *Il1b* mRNA decay in this myeloid cell type. To directly test this, WT and *TTP*^−/−^ BMDCs were stimulated for 3, 6, or 9 h with LPS, or left untreated. mRNA quantitation confirmed increased *Il1b* expression in *TTP*^−/−^ BMDCs (Supplemental Figure 3A). Comparison of mRNA stability in WT and *TTP*^−/−^ BMDCs demonstrated that the *Il1b* mRNA was destabilized by TTP prior to and 3 h after LPS stimulation (Figure 3A). Thus, the TTP-mediated acceleration of *Il1b* mRNA decay was in agreement with increased *Il1b* mRNA levels in *TTP*^−/−^ BMDCs.

We then asked whether the regulation of *Il1b* mRNA by TTP is relevant for the production of IL-1β cytokine. At the post-translational level, the bioavailability of IL-1β is controlled by proteolytic processing: *Il1b* mRNA is translated to generate an inactive 35 kDa IL-1β protein (the pro-IL-1β form) which is cleaved by the inflammasome-activated protease caspase-1 to generate the biologically active 17 kDa mature form (1). This process occurs in parallel with the mechanism necessary for IL-1β release; caspase-1 also cleaves Gasdermin-D (GSDMD), allowing the N-terminal domain to form pores in the cell plasma membrane and subsequent release of IL-1β (40).

To determine the amounts of the pro- and mature forms of IL-1β protein, BMDCs were treated with LPS alone or LPS and the inflammasome activator nigericin. Western blot analysis of lysates of these cells revealed that in the absence of TTP, both pro- and mature forms were elevated (Figure 3B and Supplemental Figure 3B). Moreover, higher IL-1β levels were detected also in the supernatants of *TTP*^−/−^ BMDCs as compared to WT controls (Figure 3C). In contrast, *TTP*^−/−^ BMDMs secreted similar IL-1β amounts as WT BMDMs (Supplemental Figure 3C), consistent with the lack of effect of TTP on *Il1b* mRNA expression in macrophages. These results indicate that the higher *Il1b* mRNA levels in *TTP*^−/−^ BMDCs caused an increase in production of pro-IL-1β and, consequently, mature IL-1β. The higher levels of both pro- and mature IL-1β suggested that processing of pro-IL-1β, hence inflammasome activity, was not regulated by TTP. In agreement, caspase-1 activity which reflects the activity of the inflammasome, was not significantly different in WT and *TTP*^−/−^ BMDCs (Figure 3D).

Together, the data demonstrate that *Il1b* mRNA is destabilized by TTP in BMDCs. The absence of TTP in these cells results in higher levels of *Il1b* mRNA which leads to increased production of mature IL-1β.

### Deletion of the IL-1 Receptor in *TTP^−/−^* Mice Delays Onset and Reduces the Severity of the TTP Deficiency Syndrome

The findings that TTP-deficient BMDCs produced more IL-1β (both mRNA and protein) (Figure 3) and our previous report showing increased *Il1b* mRNA in peritoneal neutrophils from *TTP*^−/−^ mice (37) prompted the question as to whether this level of IL-1β dysregulation is physiologically relevant. Moreover, we noticed that TTP-deficient BMDCs exhibited also higher *Il1a* mRNA expression (Supplemental Figure 4A; Supplemental Table 1) suggesting that also IL-1α dysregulation might have a physiological impact.

**Figure 4:**
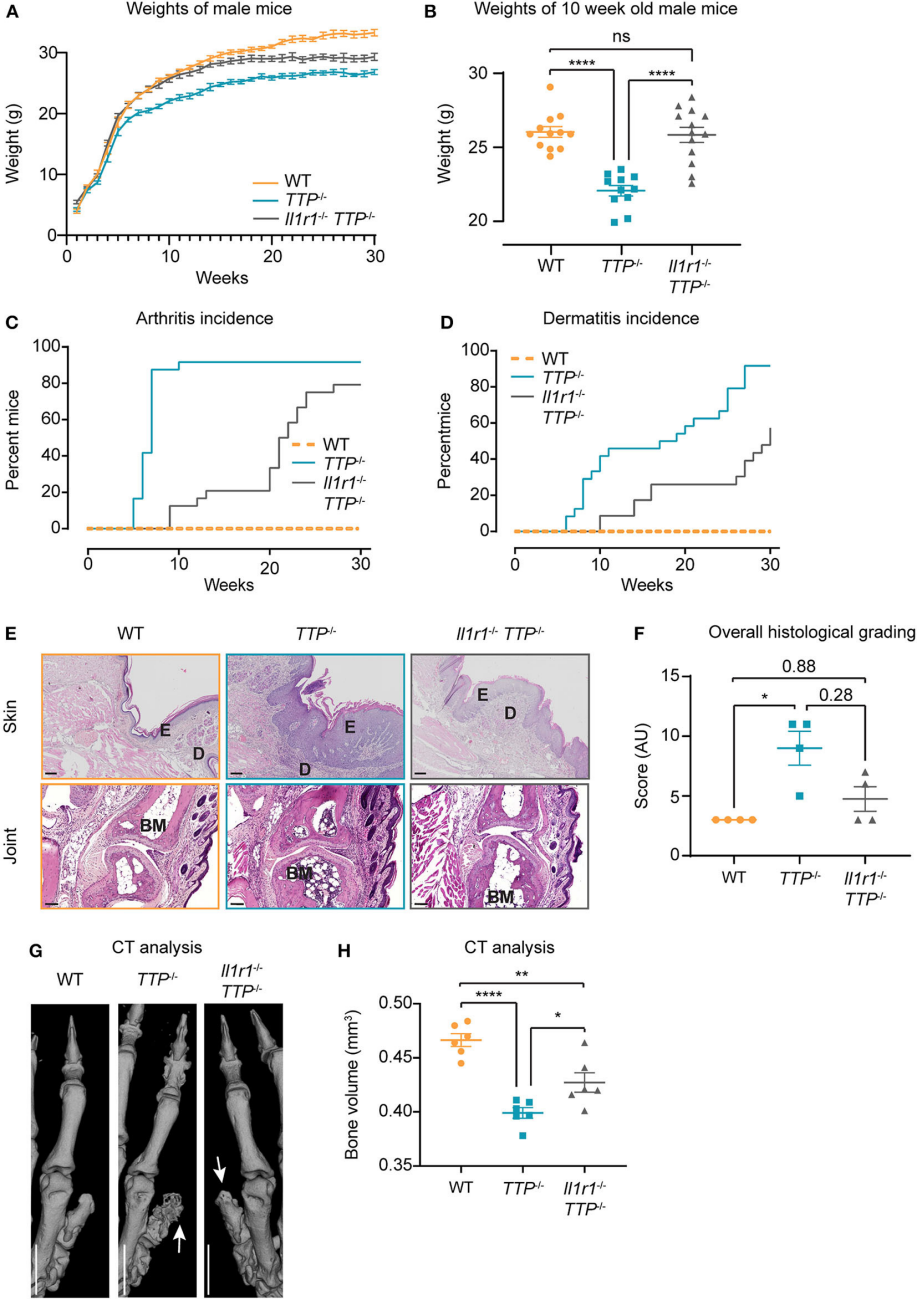
*Il1r1*^−/−^ *TTP*^−/−^ display significantly ameliorated TTP deficiency syndrome as compared to *TTP*^−/−^ mice. **(A)** Weights of WT, *TTP*^−/−^ and *Il1r1*^−/−^ *TTP*^−/−^ male mice (*n* = 12-13 per genotype), as monitored for 30 weeks. **(B)** Weights of WT, *TTP*^−/−^ and *Il1r1*^−/−^ *TTP*^−/−^ male mice (*n* = 12-13 per genotype) at 10 weeks of age. **(C)** Incidence of arthritis in male and female mice, as monitored for 30 weeks (*n* = 24 per genotype). **(D)** Incidence of dermatitis in male and female mice, as monitored for 30 weeks (*n* = 24 per genotype). **(E)** Representative H&E-stained sections of paws from WT, *TTP*^−/−^ and *Il1r1*^−/−^ *TTP*^−/−^ mice at 10 weeks of age; E, epidermis; D, dermis; BM, bone marrow. Scale bar: 100 μm. **(F)** Sections were graded for epidermal thickness, dermal infiltrate and myeloid hyperplasia. Individual scores were combined for overall grading. Statistical evaluation: Kruskal–Wallis with Dunn's multiple comparisons test; *P < 0.05. **(G)** High resolution CT imaging of front paws of 10-week-old WT, *TTP*^−/−^ and *Il1r1*^−/−^ *TTP*^−/−^ female mice. Arrows indicate pronounced bone disruption in *TTP*^−/−^ mice and weak bone disruption in *Il1r1*^−/−^ *TTP*^−/−^ mice. **(H)** Bone volume was measured in the distal phalanges (P2) from high-resolution CT scans of front paws from 10-week-old WT, *TTP*^−/−^ and *Il1r1*^−/−^ *TTP*^−/−^ mice (*n* = 6 per genotype). Scale bar: 1 mm. Statistical evaluation: one-way ANOVA with Tukey's multiple comparisons test; error bars, mean ± SEM; *P < 0.05; **P < 0.01, ****P < 0.0001.

To interrogate the possible role of IL-1 cytokines in the development of the TTP deficiency syndrome, we generated mice with a deletion in the *Il1r1* gene in *TTP*^−/−^ mice, producing *Il1r1*^−/−^ *TTP*^−/−^ mice. As expected, BMDCs from *Il1r1*^−/−^ *TTP*^−/−^ did not respond to either IL-1α or IL-1β as assessed by *Tnf* mRNA expression (Supplemental Figures 4B,C). The phenotype of these double-deficient mice was then compared to the one of *TTP*^−/−^ mice. First, we noticed that *Il1r1*^−/−^ *TTP*^−/−^ mice were able to breed (Supplemental Figure 4D), indicating an improvement in the overall health as compared to *TTP*^−/−^ mice which are not fertile presumably due to pleiotropic inflammation (23). Moreover, in contrast to the growth deficit of *TTP*^−/−^ mice, *Il1r1*^−/−^ *TTP*^−/−^ mice displayed similar growth curves as WT mice until reaching about 10 weeks of age (Figure 4A). At 10 weeks of age, both male and female *Il1r1*^−/−^ *TTP*^−/−^ mice displayed similar weights as WT mice thereby differing significantly from *TTP*^−/−^ mice which exhibited significantly lower weights (Figure 4B and Supplemental Figure 4E). At an older age, the weight of *Il1r1*^−/−^ *TTP*^−/−^ mice started to diverge from the WT but the failure to gain weight was not as severe as seen in the TTP KO mice (Figure 4A).

Accordingly, the incidence of arthritis, dermatitis and conjunctivitis were reduced in *Il1r1*^−/−^ *TTP*^−/−^ mice as compared to *TTP*^−/−^ animals (Figures 4C,D and Supplemental Figure 4F). Haematoxylin & eosin (H&E) staining of paw sections confirmed reduced inflammatory signs in *Il1r1*^−/−^ *TTP*^−/−^ mice as compared to *TTP*^−/−^ mice: *TTP*^−/−^ mice displayed thickened epidermis, severe immune cell infiltrate in the dermis and epidermis, as well as myeloid hyperplasia in the bone marrow, all of which were reduced in the *Il1r1*^−/−^ *TTP*^−/−^ mice (Figure 4E and Supplemental Figures 4G–I). The overall histological grading confirmed that inflammation was reduced (albeit not significantly) in *Il1r1*^−/−^ *TTP*^−/−^ mice as compared to *TTP*^−/−^ mice (Figure 4F). To substantiate these results, we employed high resolution computer tomography (CT) which allows the assessment of bone erosion through calculation of the bone volume. Bone disruption was detected in distal phalanges and first digits of *TTP*^−/−^ mice (Figure 4G), as previously reported (29). Importantly, the severity of bone erosion was reduced in the *Il1r1*^−/−^ *TTP*^−/−^ mice as revealed by quantitation of the bone volume (Figure 4H).

In summary, inactivation of IL-1R signaling in *TTP*^−/−^ mice results in a delayed onset and reduced severity of all aspects of the TTP deficiency syndrome, indicating that IL-1R signaling contributes to development of the hyperinflammatory phenotype.

### TTP Controls *Il1b* mRNA and IL-1β Bioavailability *in vivo*

The amelioration of the TTP deficiency syndrome observed in the *Il1r1*^−/−^ *TTP*^−/−^ mice (Figure 4) indicated that the activation of the IL-1 receptor or the signaling pathway downstream of the receptor are pathologically upregulated in *TTP*^−/−^ mice. The increased *Il1b* mRNA expression by *TTP*^−/−^ BMDCs (Figures 2, 3) and neutrophils (37) suggest that the adverse effects of the IL-1 receptor in *TTP*^−/−^ mice are caused by elevated IL-1β levels. Similarly, IL-1α might contribute to the phenotype of *TTP*^−/−^ mice as *Il1a* mRNA is increased in TTP-deficient BMDCs (Supplemental Figure 4A; Supplemental Table 1). To directly address the cause of the aberrant IL-1R function in *TTP*^−/−^ mice we conducted several types of analyses.

First, we examined whether the signaling strength downstream of the IL-1R is different in *TTP*^−/−^ vs. WT cells. To this end, WT and *TTP*^−/−^ BMDCs were stimulated with IL-1β followed by quantitation of *Tnf* gene expression as the readout for the activity of IL-1R signaling. No increased *Tnf* mRNA induction upon IL-1β treatment was detected in *TTP*^−/−^ BMDCs as compared to WT cells indicating that the signaling pathway downstream of the IL-1 receptor was not upregulated in the absence of TTP (Supplemental Figure 5A).

Second, we compared the expression of the IL-1 receptor antagonist (*Il1rn*) in *TTP*^−/−^ and WT cells. Lower *Il1rn* expression in *TTP*^−/−^ cells could result in higher stimulation of the IL-1 receptor thus increased IL-1 signaling. The BMDC RNA-Seq dataset showed no decrease in *Il1rn* expression in TTP-deficient cells (Supplemental Table 1). Similar data were observed also in BMDMs and neutrophils as reported by us previously (20, 37). This comparison indicated that the IL-1 receptor antagonist was not involved in the dysregulated function of the IL-1 receptor in *TTP*^−/−^ mice.

**Figure 5:**
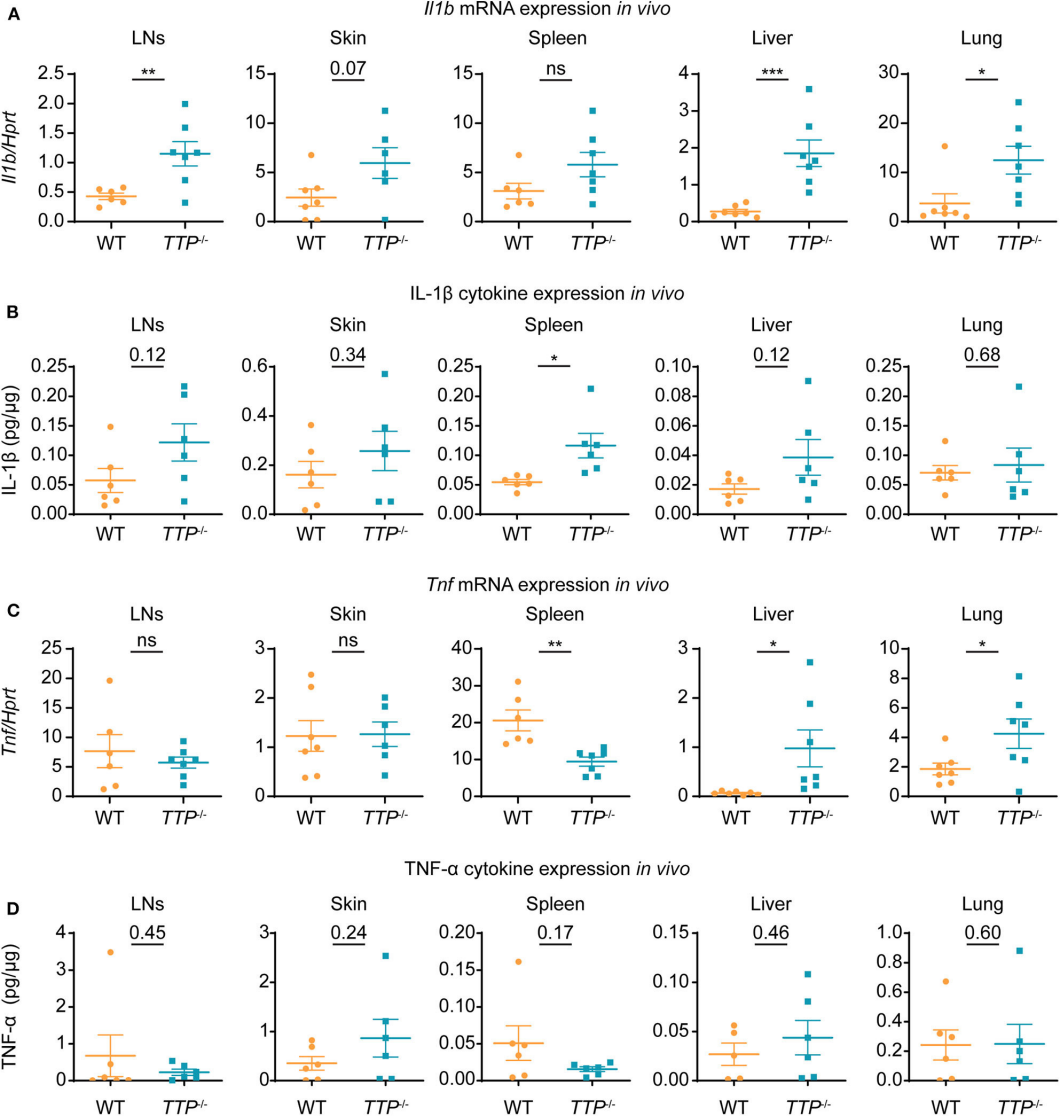
*Il1b* mRNA and IL-1β cytokine are increased in tissues of *TTP*^−/−^ mice. Mesenteric lymph nodes (LNs), skin, spleen, liver and lung were collected from 10-week-old WT and *TTP*^−/−^ mice and mRNA and cytokine levels were determined. **(A)** *Il1b* mRNA levels were quantitated by qRT-PCR (*n* = 6-7 per genotype). **(B)** IL-1β cytokine levels were measured by ELISA, with normalization to total protein (*n* = 6 per genotype). **(C,D)** *Tnf* mRNA **(C)** (*n* = 6-7 per genotype) and TNF-α cytokine **(D)** (*n* = 5-6 per genotype) were quantitated as described in **(A,B)**, respectively. Statistical evaluation: unpaired student's *t*-test; error bars, mean ± SEM; *P < 0.05; **P < 0.01; ***P < 0.001; ns, not significant.

The increased expression of *Il1a* and *Il1b* and no aberrance in signaling downstream of IL-1R in cells deficient in TTP suggested that the pathological activity of the IL-1 pathway in *TTP*^−/−^ mice was likely caused by elevated *Il1a* and/or *Il1b* expression *in vivo*. To examine whether the increased *Il1a* and *Il1b* expression in various TTP-deficient primary cells was reflected by corresponding changes in tissues, we isolated liver, lung, mesenteric lymph nodes (LNs), skin and spleen from WT and *TTP*^−/−^ mice. *Il1b* expression was significantly higher in the liver, lung and LNs from TTP-deficient animals as compared to WT controls (Figure 5A). The IL-1β cytokine was upregulated in the spleen of *TTP*^−/−^ mice and increased, albeit not significantly, in other tissues (Figure 5B). *Il1a* mRNA and IL-1α cytokine levels were similar in tissues from WT and *TTP*^−/−^ mice (Supplemental Figures 5B,C). The expression of *Tnf* , the best described TTP target, was elevated in the liver and lung but decreased in the spleen of *TTP*^−/−^ mice (Figure 5C). TNF-α cytokine was not upregulated in tissues from *TTP*^−/−^ mice (Figure 5D), consistent with previous reports (26, 41).

**Figure 6:**
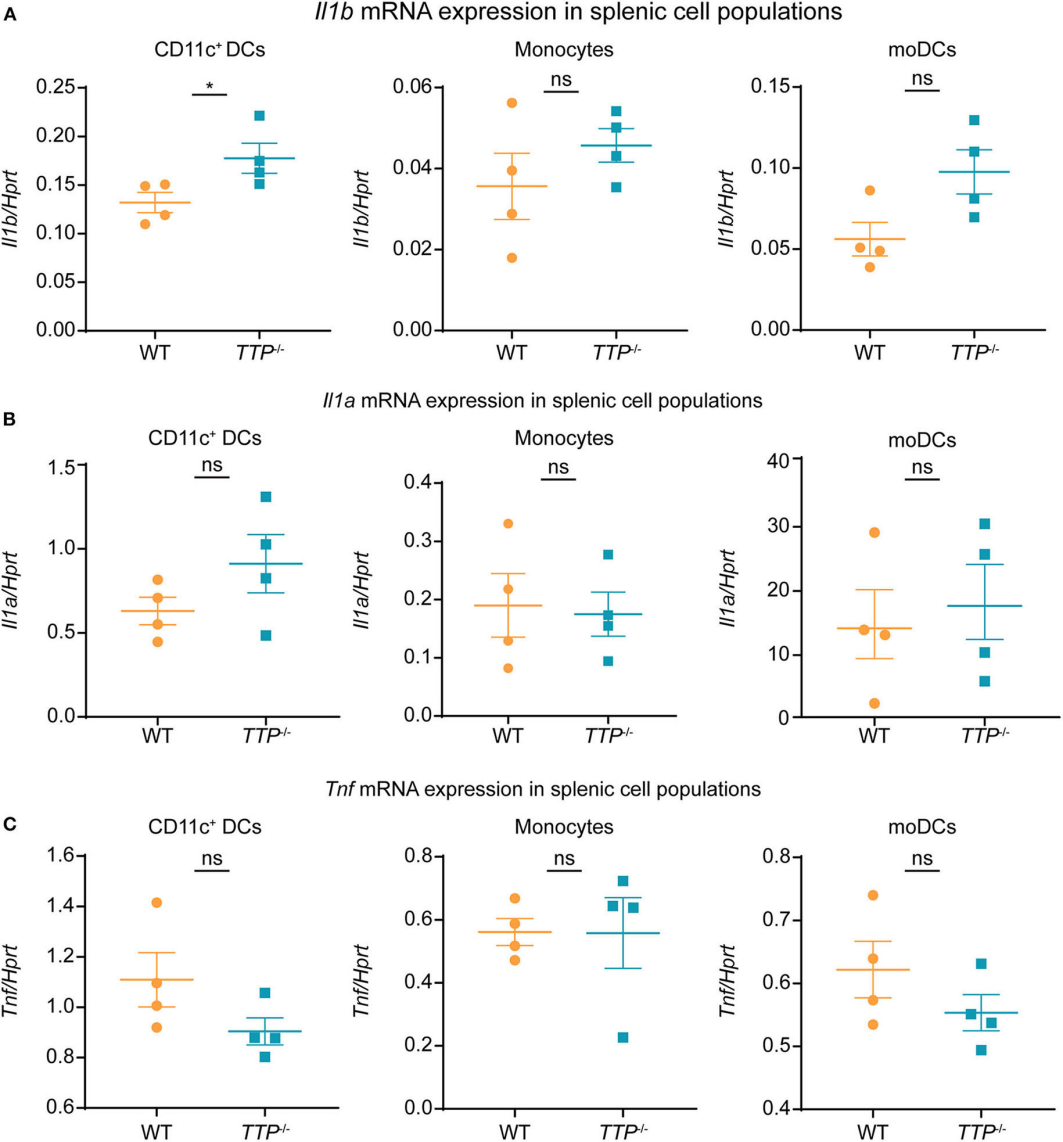
TTP controls *Il1b* expression in splenic DCs. *Il1b* **(A)**, *Il1a* **(B)**, and *Tnf* **(C)** mRNA levels were determined by qRT-PCR in CD11c^+^DCs (comprising CD11b^+^ CD11c^+^ cDCs and pDCs), monocytes (CD11b^+^ MHCII^−^ Ly6C^high^) and moDCs (CD11b^+^ MHCII^+^ Ly6C^−^ monocyte-derived DCs) sorted from spleens isolated from *TTP*^−/−^ and WT mice (*n* = 4/genotype). Sorting and gating were performed as described in Supplemental Figure 6. Statistical evaluation: unpaired student's *t*-test; error bars, mean ± SEM; *P < 0.05; ns, not significant.

The analysis of organs (Figure 5) implied that TTP was capable of controlling *Il1b* expression at least in some of the cell types forming the organ tissues. We asked whether TTP had such effects also in splenic DCs. To this end, we isolated spleens from *TTP*^−/−^ and WT mice and determined *Il1a, Il1b*, and *Tnf* expression in splenic DCs (CD11b^+^ CD11c^+^ including cDC and pDCs), CD11b^+^ MHCII^−^ Ly6C^high^ monocytes and CD11b^+^ MHCII^+^ Ly6C^−^ moDCs (monocyte-derived DCs) (Figure 6 and Supplemental Figure 6). TTP deficiency caused increased *Il1b* expression in splenic DCs (Figure 6A), consistent with effects we observed in BMDCs. *Il1b* mRNA was not elevated in TTP-deficient splenic monocytes and moDCs (Figure 6A). *Il1a* and *Tnf* mRNA were not significantly regulated by TTP in any of the analyzed splenic cell types (Figures 6B,C).

These *in vivo* and *ex vivo* analyses revealed dysregulated IL-1 production in *TTP*^−/−^ mice and supported the role of uncontrolled IL-1 signaling in the development of the TTP deficiency syndrome.

### IL-1α and IL-1β Are Non-redundant Drivers of Dysregulated IL-1 Signaling in TTP-Deficient Animals

The reduced hyperinflammation observed in *Il1r1*^−/−^ *TTP*^−/−^ mice as compared to *TTP*^−/−^ animals (Figure 4) together with increased levels of *Il1b* mRNA and IL-1β protein in tissues of *TTP*^−/−^ mice (Figures 5A,B) suggested that IL-1β plays a causal role in the TTP deficiency syndrome. IL-1α might also contribute to the TTP deficiency syndrome since higher *Il1a* mRNA levels were detected in TTP-deficient BMDCs (Supplemental Figure 4A; Supplemental Table 1) and BMDMs (20). To directly address the roles of IL-1α and IL-1β in the pathology of *TTP*^−/−^ mice we generated TTP-deficient mice lacking the *Il1a* (*Il1a*^−/−^ *TTP*^−/−^) or *Il1b* (*Il1b*^−/−^ *TTP*^−/−^) genes. As expected, *Il1a* and *Il1b* genes were not expressed in *Il1a*^−/−^ *TTP*^−/−^ and *Il1b*^−/−^ *TTP*^−/−^ mice, respectively, as revealed by LPS stimulation of BMDMs derived from these mice (Supplemental Figures 7A,B).

**Figure 7:**
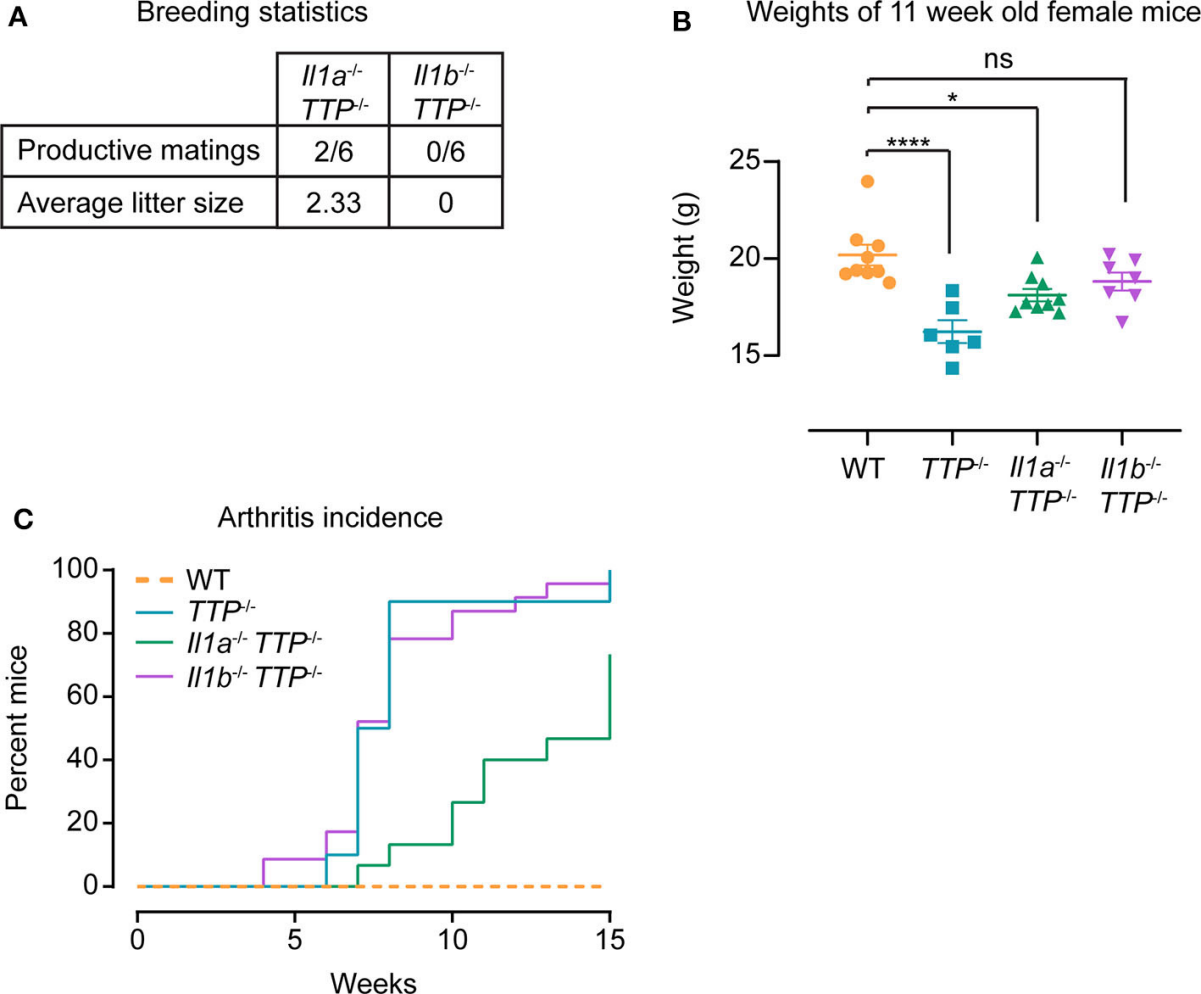
IL-1α and IL-1β contribute to TTP deficiency syndrome. WT, *TTP*^−/−^, *Il1a*^−/−^ *TTP*^−/−^, and *Il1b*^−/−^ *TTP*^−/−^ mice were phenotypically characterized for signs of the TTP deficiency syndrome. **(A)** Breeding statistics in *Il1a*^−/−^ *TTP*^−/−^ and *Il1b*^−/−^ *TTP*^−/−^ mice. Productive matings; number of females placed in breedings that had at least one live birth. **(B)** Weights of WT, *TTP*^−/−^, *Il1a*^−/−^ *TTP*^−/−^, and *Il1b*^−/−^ *TTP*^−/−^ mice at 11 weeks of age (*n* = 6–9 per genotype). Statistical evaluation: one-way ANOVA with Tukey's multiple comparisons test; error bars, mean ± SEM; *P < 0.05; ****P < 0.0001; ns, not significant. **(C)** Incidence of arthritis in WT, *TTP*^−/−^, *Il1a*^−/−^ *TTP*^−/−^, and *Il1b*^−/−^ *TTP*^−/−^ mice monitored for 15 weeks (*n* = 13–24 per genotype).

Similar to *TTP*^−/−^ mice (but in contrast to *Il1r1*^−/−^ *TTP*^−/−^ mice, Supplemental Figure 4D), *Il1b*^−/−^ *TTP*^−/−^ mice were not fertile (Figure 7A). The fertility of *Il1a*^−/−^ *TTP*^−/−^ mice was severely blunted: only 2 out of 6 breeding pairs produced viable pups (Figure 7A). The inflammatory parameters of the TTP deficiency syndrome (weight gain deficits and arthritis) were in general only slightly less pronounced in *Il1a*^−/−^ *TTP*^−/−^ and *Il1b*^−/−^ *TTP*^−/−^ mice as compared to *TTP*^−/−^ animals. At the age of 11 weeks, the weights of *Il1a*^−/−^ *TTP*^−/−^ were significantly lower than WT controls; the weights of *Il1b*^−/−^ *TTP*^−/−^ were also reduced (although not significantly) (Figure 7B). In contrast, *Il1r1*^−/−^ *TTP*^−/−^ displayed similar weights as the WT animals (Figures 4A,B). The 70 % mark of arthritis incidence was reached at 9 weeks of age in *Il1b*^−/−^ *TTP*^−/−^ mice and at 15 weeks of age in *Il1a*^−/−^ *TTP*^−/−^ mice (Figure 7C). In contrast, the same level of arthritis incidence was observed at the age of 6-7 weeks in *TTP*^−/−^ mice (Figures 4C, 7C) and at the age of 25 weeks in *Il1r1*^−/−^ *TTP*^−/−^ mice (Figure 4C).

In conclusion, these data indicate that IL-1α and IL-1β are non-redundant drivers of the TTP deficiency syndrome. The amelioration of the TTP deficiency syndrome observed in *Il1r1*^−/−^ *TTP*^−/−^ mice is due to the absence of signaling triggered by both IL-1α and IL-1β. However, the comparison of *Il1a*^−/−^ *TTP*^−/−^ and *Il1b*^−/−^ *TTP*^−/−^ mice suggests that IL-1α might contribute more to the lack of fertility and to arthritis, whereas IL-1β plays a more critical role in the weight gain deficit.

## Discussion

Despite the very high number of mRNAs bound by TTP in cells, the function of this mRNA-destabilizing protein is remarkably specific, i.e., it is mainly required for the control of immune responses (17, 18, 20–22). This functional specificity distinguishes TTP from the closely related family members *Zfp36l1* and *Zfp36l2* which are involved in various processes, including embryonic development and hematopoiesis (42–45). The preferred sequence of the target RNA is similar for all three family members and defined by an RNA binding domain composed of tandem CCCH class zinc fingers (17). Thus, the specific function of TTP is determined largely by mechanisms other than RNA binding; these mechanisms remain incompletely understood. In this study we establish that TTP can destabilize a target mRNA, namely *Il1b* RNA, in cell type-dependent ways and that such context-determined TTP function is physiologically relevant. These findings imply that TTP action is defined by the cellular context; this type of regulation could contribute to the high specificity of TTP function.

The phenotype of *TTP*^−/−^ mice, i.e., inflammation spontaneously developing after several weeks of age in many organs, including skin, joints, and eyes, has long been regarded to be caused by overexpression of *Tnf* owing to increased *Tnf* mRNA stability (46). In agreement, deletion of the TNF-α receptor *Tnfr1* in *TTP*^−/−^ mice abolished most of the TTP deficiency syndrome (with notable exception of splenomegaly which was present also upon *Tnfr1* deletion) (41).

However, one important aspect of the *TTP*^−/−^ phenotype remained unexplained: *TTP*^−/−^ mice do not develop spontaneous intestinal inflammation. This was surprising given that mice bearing deletion of the ARE sequence (i.e., the TTP binding site) in the *Tnf* gene locus suffer from severe colitis caused by *Tnf* overexpression (25). The missing signs of colitis in *TTP*^−/−^ mice indicated that also mechanisms other than *Tnf* overexpression are involved in the TTP deficiency syndrome. The findings that deletion of *Ccl3* or *Il23* in *TTP*^−/−^ mice significantly improved or restored, respectively, the health of TTP-deficient animals confirmed that *Tnf* is not an exclusive driver of the TTP deficiency phenotype (24, 47).

Our study describes IL-1 signaling triggered by IL-1α or IL-1β as an additional driver of the TTP deficiency syndrome. This finding is based on multiple lines of evidence. First, *Il1r1*^−/−^ *TTP*^−/−^ mice exhibit significantly reduced inflammatory symptoms as compared to *TTP*^−/−^ animals. Second, *Il1b* mRNA expression is increased in tissues of *TTP*^−/−^ mice and cultured primary cells from *TTP*^−/−^ mice; these cells include BMDCs shown in this study and neutrophils reported in our previous work (37). The increased *Il1b* mRNA expression causes higher levels of the mature IL-1β cytokine in tissues of *TTP*^−/−^ mice. This is important since IL-1β levels are significantly regulated by post-translational processing so that higher *Il1b* mRNA levels may not necessarily cause higher production of the mature cytokine. Increased *Il1b* mRNA was reported also for TTP-deficient keratinocytes (29). Third, the levels of *Il1a* mRNA are also elevated in TTP-deficient BMDCs (this study) and BMDMs (20).

The amelioration of the TTP deficiency syndrome by a gene deletion (i.e., *Il23, Ccl3, Tnfr1*) or antibody-mediated neutralization of a TTP target (i.e., anti-TNF-α antibodies) in *TTP*^−/−^ mice is regarded as an important evidence for a causal role of the particular gene in development of the TTP phenotype (23, 24, 41, 47). However, the precise assessment of whether a gene is directly or indirectly driving the TTP phenotype is challenging as the putative driver might be itself regulated by another TTP target. Alternatively, pathways downstream of the putative driver might be controlled by TTP.

We explored these options and their possible involvement in the driver function of Il-1 cytokines/signaling in multiple ways. First, the output of the pathway downstream of the IL-1 receptor is not changed upon TTP deletion as *Tnf* mRNA induction by IL-1α or IL-1β is similar in WT and *TTP*^−/−^ cells. Second, caspase-1 activity was not significantly different in WT and *TTP*^−/−^ BMDCs excluding a dysregulation of pro-IL-1β cleavage into mature cytokine. Third, the expression of the IL-1 receptor antagonist, a key modulator of IL-1 signaling, is not reduced in *TTP*^−/−^ cells. These control experiments together with increased *Il1a* and *Il1b* expression in *TTP*^−/−^ cells and/or tissues suggest that IL-1 signaling is a direct driver of the TTP deficiency syndrome. The incomplete rescue of the TTP deficiency syndrome by the IL-1 receptor deletion indicates that IL-1 signaling is a less robust driver as compared to the previously identified drivers TNF-α and IL-23 (23, 24).

The relatively small improvement of health of *Il1a*^−/−^ *TTP*^−/−^ and *Il1b*^−/−^ *TTP*^−/−^ mice as compared to *TTP*^−/−^ mice contrasts the significantly better health condition of *Il1r1*^−/−^ *TTP*^−/−^ mice. This finding implies that both IL-1α and IL-1β drive the TTP deficiency syndrome. IL-1α and IL-1β are effectors and amplifiers of responses to various challenges such as infections or tissue injury (1). Remarkably little is known about IL-1α and IL-1β functions under steady state (unchallenged) conditions. While IL-1β appears to facilitate glucose uptake by increasing post-prandial insulin production (48), no steady state function has been so far identified for IL-1α. Our results indicate that both IL-1α and IL-1β need to be controlled at the level of mRNA stability under steady state conditions in order to prevent perturbations in homeostasis. Although our study revealed that TTP controls *Il1a* and *Il1b* expression in DCs (BMDCs and splenic CD11c^+^ DCs) but not BMDMs and splenic monocytes, this particular TTP function cannot alone cause the TTP deficiency syndrome since mice with DC-specific TTP deletion (TTP^fl/fl^-CD11cCre mice) are healthy (29). Thus, it is likely that DCs together with other cell types drive the *Il1b*-dependent TTP deficiency syndrome. Consistent with this assumption are our findings that *Il1b* is elevated in several organs of *TTP*^−/−^ mice: these data indicate that TTP controls *Il1b* more broadly and probably also in other cells than DCs in these organs. The absence of TTP effects on *Il1a* expression in the investigated organs suggests that TTP controls this cytokine in small cell subsets in these organs and/or that *Il1a* is significantly regulated by TTP in cells from other organs. Regardless of the identity of the most relevant cell types for the *Il1a*- and *Il1b*-driven TTP deficiency syndrome, our study establishes the critical importance of cell-type dependent control of TTP-mediated mRNA destabilization *in vivo*.

Previous reports by us and others suggested the involvement of cell type-specific factors in TTP activity: while *Il1b* mRNA was expressed in similar amounts in WT and *TTP*^−/−^ BMDMs, the levels of this mRNA were higher in *TTP*^−/−^ neutrophils and keratinocytes as compared to WT controls (20, 29, 37). However, the evidence that these differences were caused by higher *Il1b* mRNA stability in specific *TTP*^−/−^ cells was not provided. The cell type-specific effects of TTP are not restricted to *Il1b* but comprise many TTP targets as revealed by the significant differences of TTP effects on the transcriptomes of BMDCs vs. BMDMs shown in this study, and BMDMs vs. neutrophils reported previously (37). The mechanisms underlying this context-dependent mRNA destabilization remain to be discovered in future studies. These mechanisms might include cell type-specific auxiliary factors that modulate (positively or negatively) the destabilizing activity of TTP for a particular target (or a group of targets). Cell type-specific post-translational modification of TTP may also contribute to the context-dependent function of TTP. Recent studies revealed that TTP contains a large number of phosphorylation sites with so far unexplained biological functions (39, 49).

Regulated mRNA degradation is hallmark of controlled immune responses (15, 50). The principal regulated parameters are selectivity and timing of mRNA decay. Our current study reveals another important level of regulation, namely context-dependent destabilization of mRNA as key process in the maintenance of immune homeostasis.

## Data Availability Statement

The datasets generated for this study can be found in the RNA-Seq data were deposited in GEO under the accession number GSE143241.

## Ethics Statement

The animal study was reviewed and approved by Austrian Ministry of Science; license number: BMWFW-66.006/0019-WF/V/3b/2016.

## Author Contributions

LS and PK conceptualized, designed the experiments, and wrote the manuscript. LS established experimental systems, conducted and analyzed RNA-Seq, performed phenotypic analysis of mice, and analyzed primary cells *in vitro*. KE designed and analyzed *in vivo* systems. HD performed IL-1β cytokine analysis *in vitro*. VS analyzed RNA-Seq. AL analyzed *Il1b* mRNA *in vitro*. FE established *Il1r1*^−/−^ *TTP*^−/−^ mice. IF generated tissue sections. YI generated *Il1a* ^−/−^ mice. All authors contributed to data interpretation.

## Conflict of Interest

The authors declare that the research was conducted in the absence of any commercial or financial relationships that could be construed as a potential conflict of interest.
