# Supplementary material for: Context-Dependent IL-1 mRNA-Destabilization by TTP Prevents Dysregulation of Immune Homeostasis Under Steady State Conditions
Source: Front Immunol. 2020 Jul 7;11:1398. doi: 10.3389/fimmu.2020.01398 (PMC7358311; doi:10.3389/fimmu.2020.01398)

Context-dependent IL-1 mRNA-destabilization by TTP prevents dysregulation of immune homeostasis under steady state conditions

Lucy Sneezum, Kevin Eislmayr, Helene Dworak, Vitaly Sedlyarov, Anita Le Heron, Florian Ebner, Irmgard Fischer, Yoichiro Iwakura and Pavel Kovarik

SUPPLEMENTAL MATERIAL: Supplemental Figure Legends and Figures

Supplemental Figure Legends:

Supplemental Figure 1 (related to Figure 1). TTP expression and MK2 phosphorylation in BMDCs and quality control of RNA-Seq

(**A**) Western blot analysis using whole cell extracts confirms that BMDCs from *TTP*$^{-/-}$ mice do not express TTP protein. Arrows point to TTP and unspecific (Unsp.) protein bands. Tubulin served as loading control. (**B**) Principal component (PC) analysis of biological triplicates from RNA-Seq experiment described in Figure 1 (BMDCs from WT and *TTP*$^{-/-}$ mice; 0, 3, 6 and 9 h LPS). (**C**) Western blot analysis of TTP protein expression during LPS stimulation of BMDCs and BMDMs. (**D**) Western blot analysis of MK2 phosphorylation (p-MK2) BMDMs and BMDCs stimulated for 3, 6 and 9 h with LPS or left untreated. MK2 and tubulin served as loading controls.

Supplemental Figure 2 (related to Figure 2). TTP binds to Il1b 3' UTR in BMDMs

TTP binding site on *Il1b* mRNA in BMDMs as determined by PAR-iCLIP reported by us previously [20] and visualized using TTP Atlas. TSS, transcription start site; nt, nucleotides.

Supplemental Figure 3 (related to Figure 3). TTP controls *Il1b* expression BMDCs but not BMDMs

(**A**) *Il1b* mRNA levels in *TTP*$^{-/-}$ and WT BMDCs stimulated for 0, 3, 6 and 9 h with LPS, as determined by qRT-PCR with normalization to *Hprt*. (**B**) Quantification (using Bio-Rad Image Lab) of Western blot shown in Figure 3B; pro-IL-1β (left panel) and mature IL-1β (right panel) were quantified with normalization to tubulin. (**C**) Secreted levels of IL-1β measured by ELISA using supernatants from *TTP*$^{-/-}$ and WT BMDMs stimulated for 0, 3 and 6 h with LPS in the

presence or absence of nigericin, as indicated. Statistical evaluation in (A) and (C): two-way ANOVA with Sidak's multiple comparisons test; error bars, mean ± SEM; ***, $P < 0.001$; ****, $P < 0.0001$; ns, not significant.

Supplemental Figure 4 (related to Figure 4). IL-1 signaling contributes to TTP deficiency syndrome

(**A**) RNA-Seq data (FPKM) for *Il1a* mRNA in *TTP*$^{-/-}$ and WT BMDCs stimulated for 0, 3, 6 and 9 h with LPS. (**B, C**) WT and *Il1r1*$^{-/-}$ *TTP*$^{-/-}$ BMDCs were left untreated (0 h) or treated with IL-1α (B) or IL-1β (C) for 6 h followed by quantification of *Tnf* mRNA levels using qRT-PCR. Note that IL-1α and IL-1β increased *Tnf* expression in WT but nor *Il1r1*$^{-/-}$ *TTP*$^{-/-}$ BMDCs. Statistical evaluation in (A), (B) and (C): two-way ANOVA with Sidak's multiple comparisons test; error bars, mean ± SEM; *, $P < 0.05$; **, $P < 0.01$, ***, $P < 0.001$; ****, $P < 0.0001$; ns, not significant. (**d**) Breeding statistics for *TTP*$^{-/-}$ and *Il1r1*$^{-/-}$ *TTP* $^{-/-}$ mice as compared to *TTP*$^{+/-}$ mice which breed as WT mice. Productive matings: number of females placed in breedings that had at least one live birth. (**E**) Weights of female WT, *TTP* $^{-/-}$ and *Il1r1*$^{-/-}$ *TTP* $^{-/-}$ mice at 10 weeks of age (n = 12-14 per genotype). (**F**) Incidence of conjunctivitis in male and female WT, *TTP* $^{-/-}$ and *Il1r1*$^{-/-}$ *TTP* $^{-/-}$ mice, as monitored for 30 weeks (n = 24 per genotype). (**G**) Degree of splenomegaly in WT, *TTP* $^{-/-}$ and *Il1r1*$^{-/-}$ *TTP* $^{-/-}$ mice (10 weeks old), as determined by normalizing spleen weight to body weight (n = 5-6 per genotype). Statistical evaluation in (E) and (G): one-way ANOVA with Tukey's multiple comparisons test; error bars, mean ± SEM; ****, $P < 0.0001$; ns, not significant. (**H, I** and **J**) H&E-stained sections of paws shown in Figure 4E were graded for epidermal thickness (H), dermal infiltration (I) and myeloid hyperplasia in the bone marrow (J). Statistical evaluation: Kruskal-Wallis test with Dunn's multiple comparisons test; *, $P < 0.05$; other values are as indicated.

Supplemental Figure 5 (related to Figure 5). *Il1a* mRNA and IL-1α cytokine in tissues of *TTP*$^{-/-}$ mice

(**A**) WT and *TTP*$^{-/-}$ BMDCs were treated with 10 ng/ml IL-1β for 0, 6 and 9 h followed by quantitation of *Tnf* mRNA using qRT-PCR. Statistical evaluation: two-way ANOVA with Sidak's multiple comparisons test; error bars, mean ± SEM; *, $P < 0.05$; ns, not significant. (**B, C**) Mesenteric lymph nodes (LNs), skin, spleen, liver and lung were collected from 10-week-old WT and *TTP* $^{-/-}$ mice and *Il1a* mRNA (B) and IL-1α cytokine (C) levels were determined by qRT-PCR and ELISA, respectively. n = 6-7 per genotype. Statistical evaluation in (B) and (C): unpaired student's t-test; error bars, mean ± SEM; ns, not significant.

Supplemental Figure 6 (related to Figure 6). Sorting scheme of splenic cell populations for analysis of DCs, monocytes and moDCs

(**A**) Splenic cells were harvested and stained as described in Methods. Debris (SSC-A/FSC-A), doublets (FSC-A/FSC-H and SSC-A/SSC-H) and dead cells (Fixable Viability Dye) were excluded. B cells and T cells were removed as $B220^+$ and $CD3^+$ cells. $CD11c^+$-DCs (including $CD8^+$ and $CD8^-$ cDCs as well as pDCs) were identified as $CD11c^+$ and $CD11b^+$. $CD11b^+$ $MHCII^-$ $Ly6C^{high}$ monocytes and $CD11b^+$ $MHCII^+$ $Ly6C^-$ moDC (monocyte-derived DCs) were selected from $CD11b^+$ subpopulation and separated from eosinophils using the side scatter height. (**B**) Purity of the analyzed subsets. FlowJo_V10.6.1 software was used for analysis and visual representation.

Supplemental Figure 7 (related to Figure 7). BMDMs from *Il1a*$^{-/-}$ *TTP*$^{-/-}$ and *Il1b*$^{-/-}$ *TTP*$^{-/-}$ mice do not express *Il1a* and *Il1b*, respectively

(**A**) *TTP*$^{-/-}$ and *Il1a*$^{-/-}$ *TTP*$^{-/-}$ BMDMs were left untreated (0 h) or treated with LPS for 6 h followed by quantitation of *Il1a* mRNA using qRT-PCR with normalization to *Hprt*. (**B**) *TTP*$^{-/-}$ and *Il1b*$^{-/-}$ *TTP*$^{-/-}$ BMDMs were left untreated (0 h) or treated with LPS for 6 h followed by quantitation of *Il1b* mRNA using qRT-PCR with normalization to *Hprt*. Statistical evaluation: two-way ANOVA with Sidak's multiple comparisons test; error bars, mean ± SEM; ****, $P < 0.0001$; ns, not significant.

Supplemental Figures:

Supplemental Figure 1

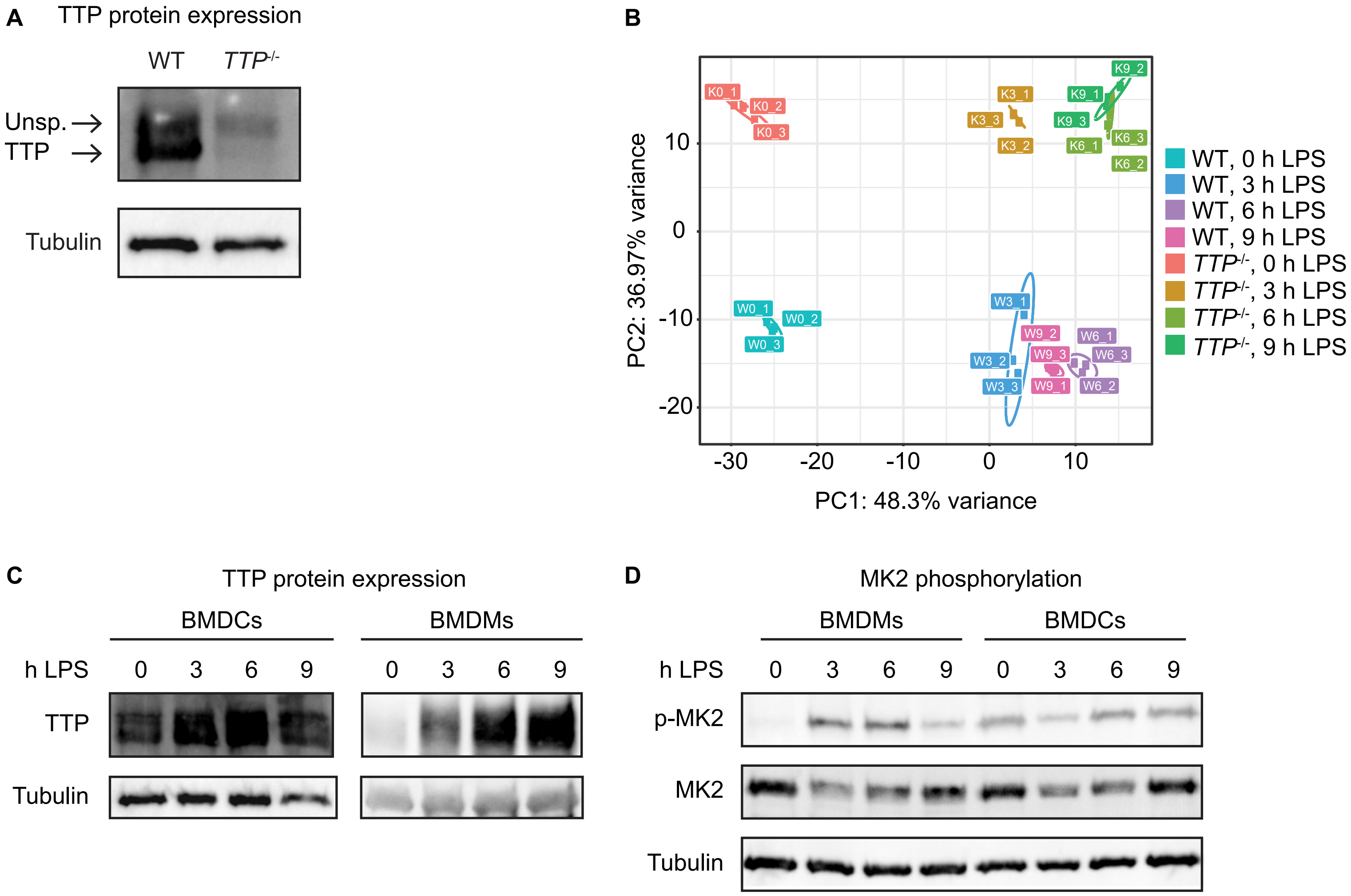

Supplemental Figure 2

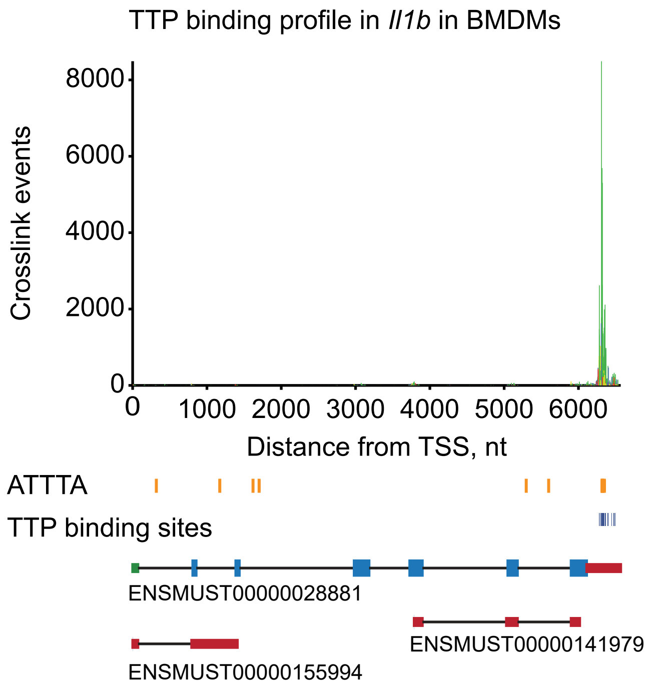

Supplemental Figure 3

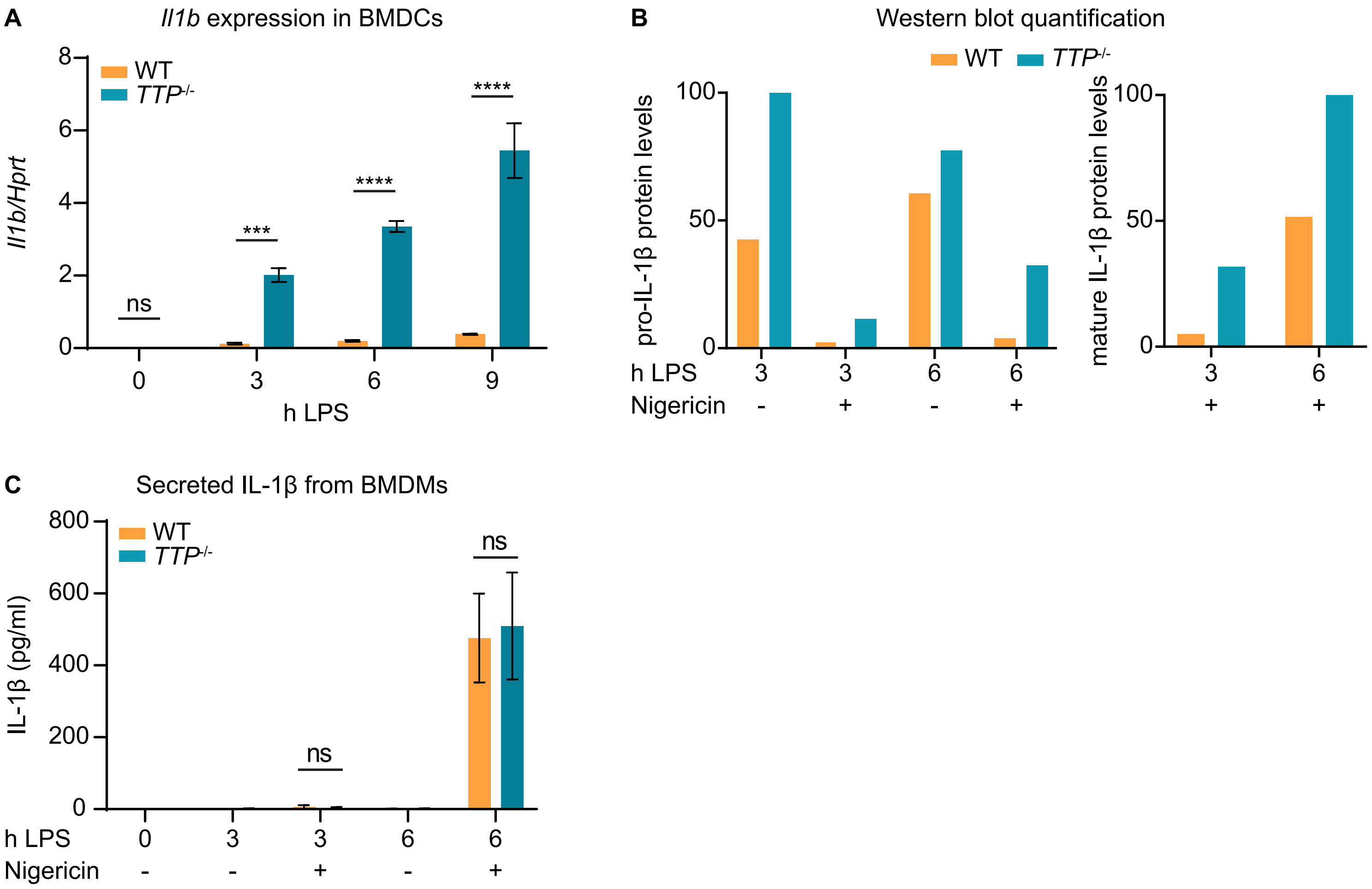

Supplemental Figure 4

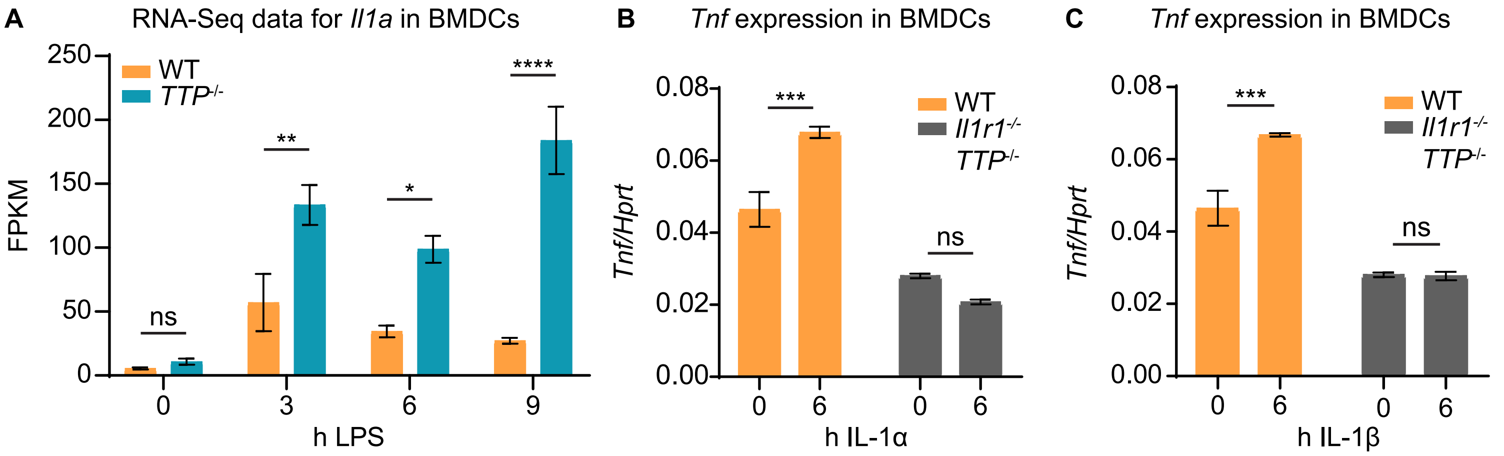

D Breeding statistics

	TTP+/-	*TTP*-/-	*Il1r1*-/- *TTP*-/-
Productive matings	10/10	0/7	8/10
Average litter size	6.66	0	3.67

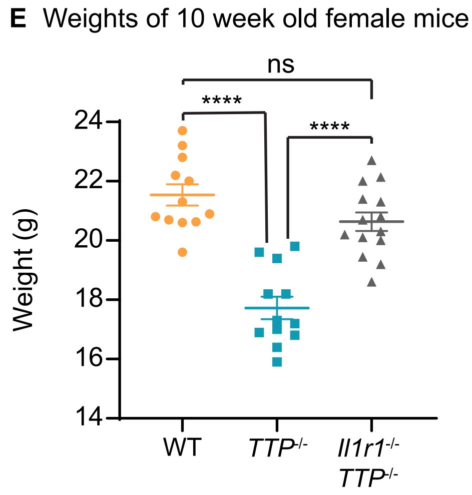

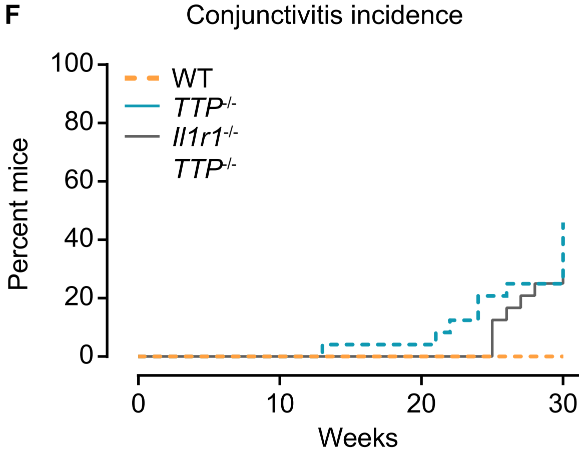

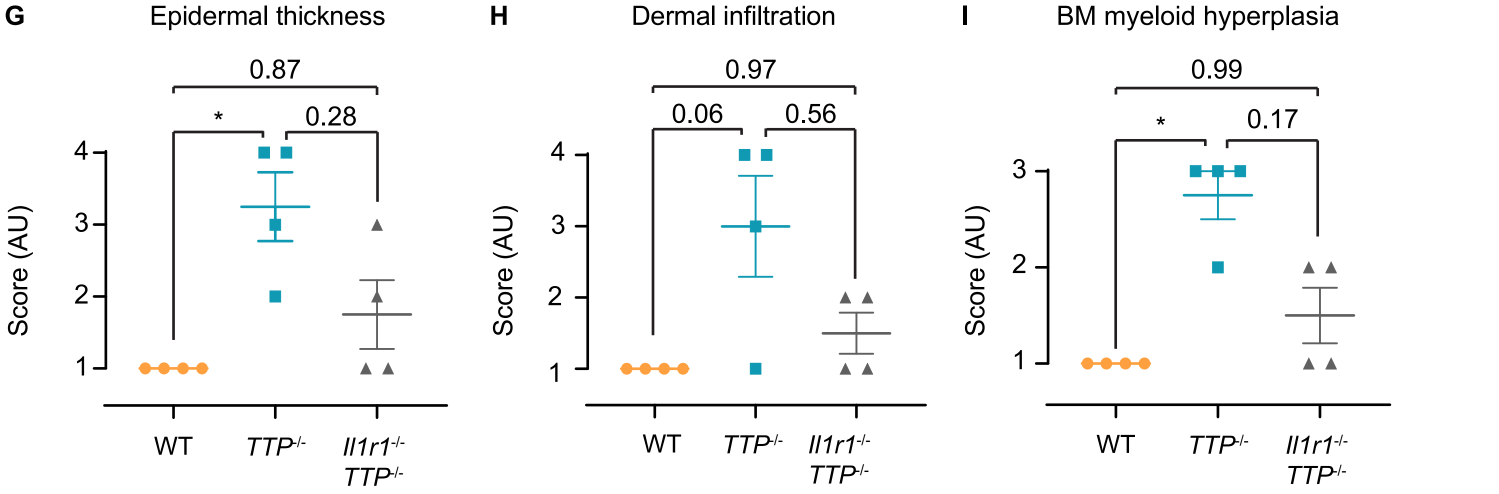

Supplemental Figure 5

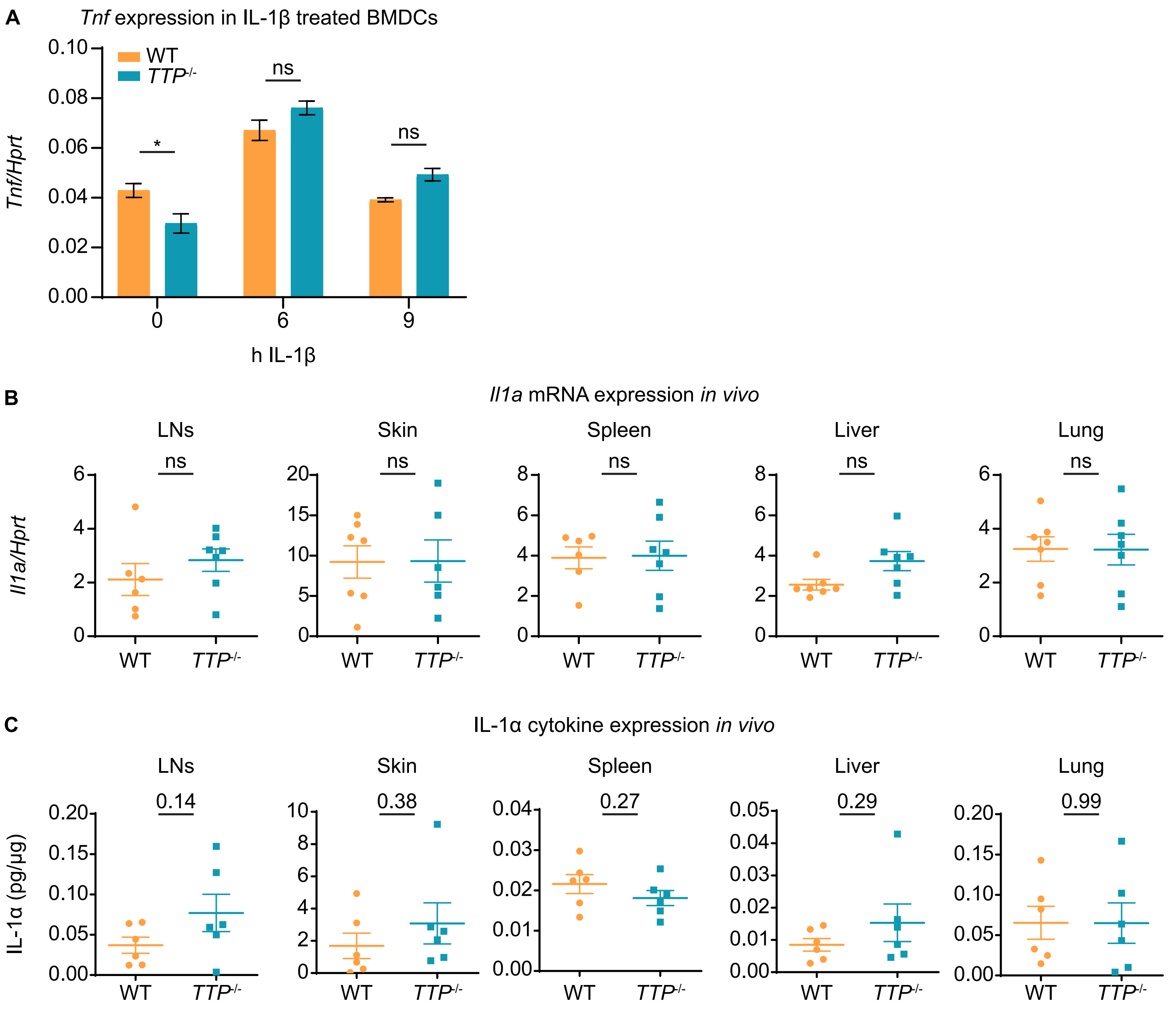

Supplemental Figure 6

A Flow cytometry gating strategy

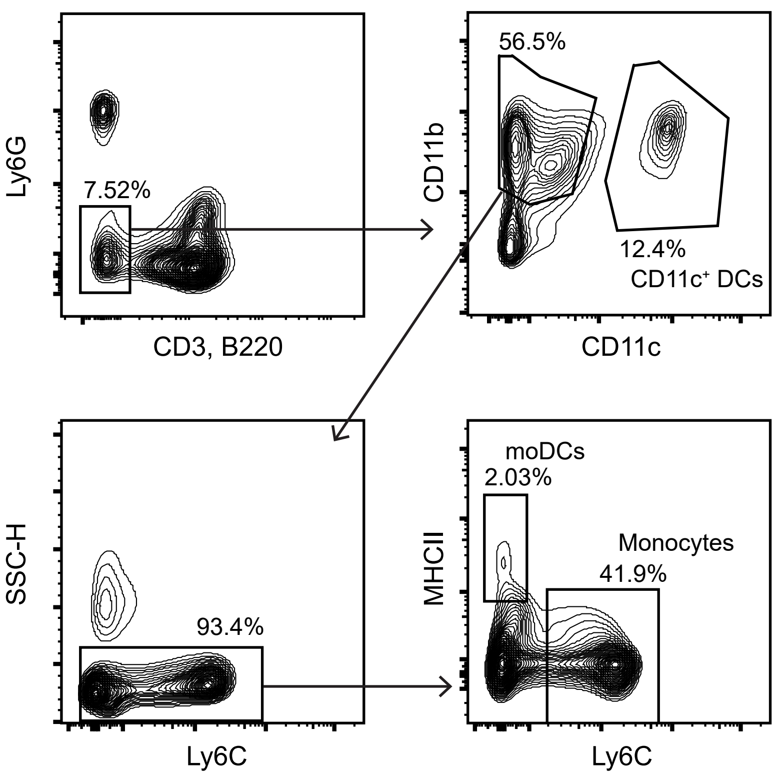

B Cell population purity

Sorted population	% of alive
CD11c⁺ DCs	79 - 83
Monocytes	75 - 78
moDCs	64 - 69

Supplemental Figure 7

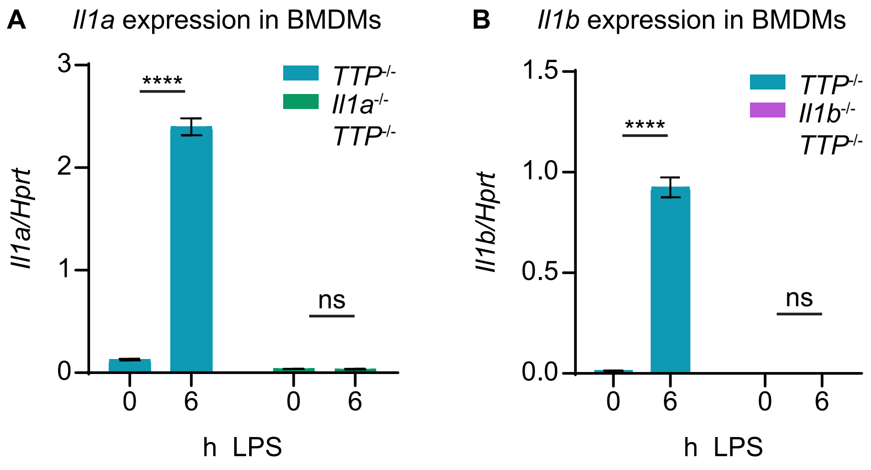

Supplement: Supplementary file 1 [file Data_Sheet_1.pdf]
